# Relationship between Translational and Rotational Dynamics of Alkyltriethylammonium-Based Ionic Liquids

**DOI:** 10.3390/ijms23031688

**Published:** 2022-02-01

**Authors:** Danuta Kruk, Elzbieta Masiewicz, Sylwia Lotarska, Roksana Markiewicz, Stefan Jurga

**Affiliations:** 1 Department of Physics and Biophysics, University of Warmia & Mazury in Olsztyn, Oczapowskiego 4, 10-719 Olsztyn, Poland; elzbieta.masiewicz@uwm.edu.pl (E.M.); sylwia.lotarska@uwm.edu.pl (S.L.); 2 NanoBioMedical Centre, Adam Mickiewicz University, Poznan, Wszechnicy Piastowskiej 3, 61-614 Poznan, Poland; roksana.markiewicz@amu.edu.pl (R.M.); stjurga@amu.edu.pl (S.J.)

**Keywords:** ionic liquids, dynamics, diffusion, relaxation, nuclear magnetic resonance

## Abstract

^1^H spin-lattice relaxation experiments have been performed for a series of ionic liquids including bis(trifluoromethanesulfonyl)imide anion and cations of a varying alkyl chain length: triethylhexylammonium, triethyloctylammonium, decyltriethylammonium, dodecyltriethylammonium, triethyltetradecylammonium, and hexadecyltriethylammonium. The relaxation studies were carried out in abroad frequency range covering three orders of magnitude, from 10 kHz to 10 MHz, versus temperature. On the basis of a thorough, quantitative analysis of this reach data set, parameters characterizing the relative, cation-cation, translation diffusion (relative diffusion coefficients and translational correlation times), and rotational motion of the cation (rotational correlation times) were determined. Relationships between these quantities and their dependence on the alkyl chain length were discussed in comparison to analogous properties of molecular liquids. It was shown, among other findings, that the ratio between the translational and rotational correlation times is smaller than for molecular liquids and considerably dependent on temperature. Moreover, a comparison of relative and self-diffusion coefficients indicate correlated translational dynamics of the cations.

## 1. Introduction

One of the fundamental questions of condensed matter science concerns dynamical properties of molecular and ionic liquids. Various aspects of this subject are considered—one asks, among other questions, about the mechanisms of translational and rotational motion, relationships between different dynamical processes, the influence of electrostatic interactions on the molecular and ionic motion (molecular liquids versus ionic liquids), correlation, and cooperativity effects.

Nuclear Magnetic Resonance (NMR) methods give a highly valuable insight into dynamical properties of liquids. One of the methods is referred to as NMR diffusometry—molecules or ions including NMR active nuclei (typically ^1^H or ^19^F) move in a magnetic field gradient. As a result of the translation diffusion, one observes changes in the resonance frequency of the nuclei originating from changes in the magnetic field at different locations in space [1,2]. This description of NMR diffusometry is simplified, but it captures the essential concept. In this way, one measures self-diffusion coefficients of molecules and ions. Another method of enquiring into dynamical properties of liquids is NMR relaxometry. In this case, the information about the dynamics is encoded into quantities referred to as relaxation times, mostly the spin-lattice relaxation time (its reciprocal value is called spin-lattice relaxation rate). The principle of the relaxation process is as follows. In an external magnetic field, there are two available energy levels for nuclei of the spin quantum number 1/2 (such as ^1^H or ^19^F)—the energy levels correspond to parallel and anti-parallel orientations of the magnetic moment of the nucleus with respect to the direction of the external magnetic field. As according to the Boltzmann distribution, the population of the lower energy level is higher and the system gains a magnetization resulting from the difference in the populations of the two energy levels. When the magnetic field changes, the system repopulates, and the magnetization evolves in time towards the new equilibrium (determined by the Boltzmann distribution). In most cases the evolution of the magnetization is exponential, and the characteristic time constant is referred to as the spin-lattice relaxation time (in case of a non-exponential magnetization evolution, the description is more complex, but the meaning of the spin-lattice relaxation time remains unchanged). The repopulations of the energy levels require an exchange of energy with the surrounding (lattice) formed by neighboring NMR active nuclei. The pathway for the exchange is provided (in case of spin-1/2 nuclei) by magnetic dipole–dipole interactions that fluctuate in time as a result of molecular (ionic) motion. The dipole–dipole interactions can be of inter-molecular (inter-ionic) or intra-molecular (intra-ionic) origin; the first one fluctuate in time as a result of translation diffusion, while the second one is modulated by rotational dynamics. The relaxation rate depends on the amplitude of the dipole-dipole coupling, the time scale of the dynamical process responsible for stochastic time fluctuations of the dipole-dipole coupling (the characteristic time constant is referred to as a correlation time), and the mechanism of the motion [3,4,5,6,7,8,9,10,11]. Consequently, NMR relaxation studies give access to the molecular (ionic) dynamics. It is, however, very important to point out that the relaxation rate reaches its maximum when the correlation time matches the reciprocal resonance frequency. This implies that at low magnetic fields (and, hence, low resonance frequencies), one probes slow dynamics, while with increasing resonance frequency, faster dynamical processes come into play. Therefore, NMR relaxation experiments carried out in a broad frequency range make it possible to probe the translational and rotational dynamics in a single experiment. This method is called NMR relaxometry [12,13] and the covered frequency range encompasses at least four orders of magnitude (from 10 kHz to 10 MHz, referring to ^1^H nuclei). NMR relaxometry has extensively been exploited to enquire into translational and rotational dynamics of molecular liquids and relationships between the two kinds of motion [14,15,16,17,18,19,20,21]. Much less studies have been performed for ionic liquids. There is, however, a growing awareness that NMR relaxometry is a very valuable source of information about ionic dynamics, both the cation and the anion, in bulk ionic liquids and in confinement [22,23,24,25,26,27,28,29]. One should also point out that Electron Spin Resonance gives a highly valuable insight into dynamical properties of ionic liquids by investigating the performance of paramagnetic probes placed in ionic liquids [30,31,32,33,34].

In this work, we exploit NMR relaxometry to inquire into dynamical properties of a series of ionic liquids: triethyloctylammonium bis(trifluoromethanesulfonyl)imide ([TEA-C8][TFSI])—C_16_H_32_F_6_N_2_O_2_S_2_, dodecyltriethylammonium bis(trifluoromethanesulfonyl)imide ([TEA-C12][TFSI])—C_20_H_40_F_6_N_2_O_2_S_2_, and hexadecyltriethylammonium bis(trifluoromethanesulfonyl)imide ([TEA-C16][TFSI])—C_24_H_48_F_6_N_2_O_2_S_2_. The liquids include the same anion, while the cations differ with respect to the length of the alkyl chain. To some extent, this work can be treated as a continuation of our previous studies [35] focused on dynamical properties of butyltriethylammonium bis(trifluoromethanesulfonyl)imide ([TEA-C4][TFSI])—C_12_H_24_F_6_N_2_O_2_S_2_. In the present work, we aim at getting insight into two subjects. The first one is to reveal how the structure of the cation influences its rotational dynamics. The second question concerns translation diffusion. Inter-molecular (inter-ionic) dipole-dipole interactions are modulated by relative translation motion. For non-correlated dynamics, the relative diffusion coefficient is equal to the sum of the self-diffusion coefficients of the interacting species; this means that for identical molecules (ions) the relative diffusion coefficient is twice larger than the self-diffusion coefficient. As already pointed out, self-diffusion coefficients can be obtained by NMR diffusometry. Then, by comparing self-diffusion and relative diffusion coefficients, one can get insight into correlation in the translation movement [35]. Such comparisons have been performed for molecular liquids [36], indicating lack of correlation, as expected. Analogous studies for ionic liquids are rare, although actually for ionic systems, one can expect a correlated translation movement. Profiting from the self-diffusion coefficients reported for the cations in these liquids in [37], obtained by means of NMR diffusometry, we enquire into the subject of correlated dynamics. Eventually, combining the two subjects, we discuss the relationship between the translational and rotational dynamics, in terms of the correlation times characterizing these processes, in comparison to molecular liquids.

## 2. Theory

^1^H relaxation processes are caused by magnetic dipole-dipole interactions that can be of intra-molecular (intra-ionic) and inter-molecular (inter-ionic) origin. Consequently, the overall ^1^H spin-lattice relaxation rate, $R_{1H}\left(\omega \right)$ (ω denotes ^1^H resonance frequency in angular frequency units) includes two contributions:

(1) $$R_{1H}\left(\omega \right)\;=R_{1H}^{intra}\left(\omega \right)+R_{1H}^{inter}\left(\omega \right)$$

where $R_{1H}^{intra}\left(\omega \right)$ and $R_{1H}^{inter}\left(\omega \right)$ denote the intra-ionic and inter-ionic relaxation contributions, respectively. The intra-ionic dipole-dipole interactions fluctuate in time due to rotational dynamics of the cation and the corresponding relaxation rate, $R_{1H}^{intra}\left(\omega \right)$, which is given as [3,4,5,6,7]:

(2) $$R_{1H}^{intra}\left(\omega \right)=C_{DD}^{HH}\left[\frac{\tau_{rot}^{C}}{1+{\left(\omega \tau_{rot}^{C}\right)}^{2}}+\frac{4\tau_{rot}^{C}}{1+{\left(2\omega \tau_{rot}^{C}\right)}^{2}}\right]$$

where $C_{DD}^{HH}$ is a dipolar relaxation constant, while $\tau_{rot}^{C}$ is referred to as a rotational correlation time (of the cation, in this case). The inter-ionic relaxation contribution, $R_{1H}^{inter}\left(\omega \right)$, is associated with relative translational diffusion of the interacting ions and can be expressed as [4,5,8,9,18]:

(3) $$R_{1H}^{inter}\left(\omega \right)=\;\frac{108}{5}{\left(\frac{\mu_{0}}{4\pi }\gamma_{H}^{2}\hbar \right)}^{2}\frac{1}{d_{CC}^{3}}N_{H}\overset{\infty }{\underset{0}{\int }}\frac{u^{4}}{81+9u^{2}-2u^{4}+u^{6}}\left[\frac{\tau_{trans}^{C}}{u^{4}+{\left(\omega \tau_{trans}^{C}\right)}^{2}}+\frac{4\tau_{trans}^{C}}{u^{4}+{\left(2\omega \tau_{trans}^{C}\right)}^{2}}\right]du$$

where the translational correlation time of the cation, $\tau_{trans}^{C}$, is defined as: $\tau_{trans}^{C}=\frac{d_{CC}^{2}}{D_{trans}^{CC}}$; $d_{CC}$ denotes the cation-cation distance of closest approach; $D_{trans}^{CC}$ is the translation relative, cation-cation translation diffusion coefficient; $N_{H}$ denotes the numbers of ^1^H nuclei per unit volume; other symbols have well-known meanings. Equation (3) assumes that the translational motion is isotropic (three-dimensional), as expected for liquids in bulk. For three-dimensional diffusion, one observes a linear dependence of the relaxation rate on a squared root of the resonance frequency, $\sqrt{\omega }$, in the low frequency range, in which the condition $\omega \tau_{trans}\ll 1$ is fulfilled [23]. The translation diffusion coefficient, $D_{trans}^{CC}$, can be obtained from the low frequency slope, b, of $R_{1H}\left(\omega \right)$ versus $\sqrt{\omega }$, from the relationship [23]:

(4) $$b=-\frac{2^{-2/3}\pi }{30}\left(1+4\sqrt{2}\right){\left(\frac{\mu_{0}}{4\pi }\gamma_{H}^{2}\hbar \right)}^{2}N_{H}{\left(D_{trans}^{CC}\right)}^{-3/2}\sqrt{\omega }\;\;\;{\;}^{}$$

## 3. Materials and Methods

^1^H spin-lattice relaxation measurements were performed for [TEA-C6][TFSI], [TEA-C8][TFSI], [TEA-C10][TFSI], [TEA-C12][TFSI], [TEA-C14][TFSI], and [TEA-C16][TFSI] in the frequency range from 10 kHz to 10 MHz versus temperature, using a NMR relaxometer, produced by Stelar s.r.l. (Mede (PV), Italy). The temperature was controlled with an accuracy of 0.5 K. The experiments started with the highest temperature, and then the temperature was progressively decreased. For each resonance frequency, 32 magnetization values were recorded versus time in a logarithmic time scale. Below 4 MHz pre-polarization at 0.19 T was applied. The switching time of the magnet was set to 3 ms. The relaxation processes turned out to be single-exponential for all temperatures in the whole frequency range for all liquids. The magnetization curves (^1^H magnetization versus time) are shown in Supplementary Materials.

The basic properties of the ionic liquids are summarized in Table 1. The synthetic procedure, as well as crystallization temperatures of the presented compounds, $T_{cryst}$, were taken from [37]. The thermodynamic parameters of the liquids are listed in Table 1 of [37].

## 4. Results and Analysis

**Figure 1:**
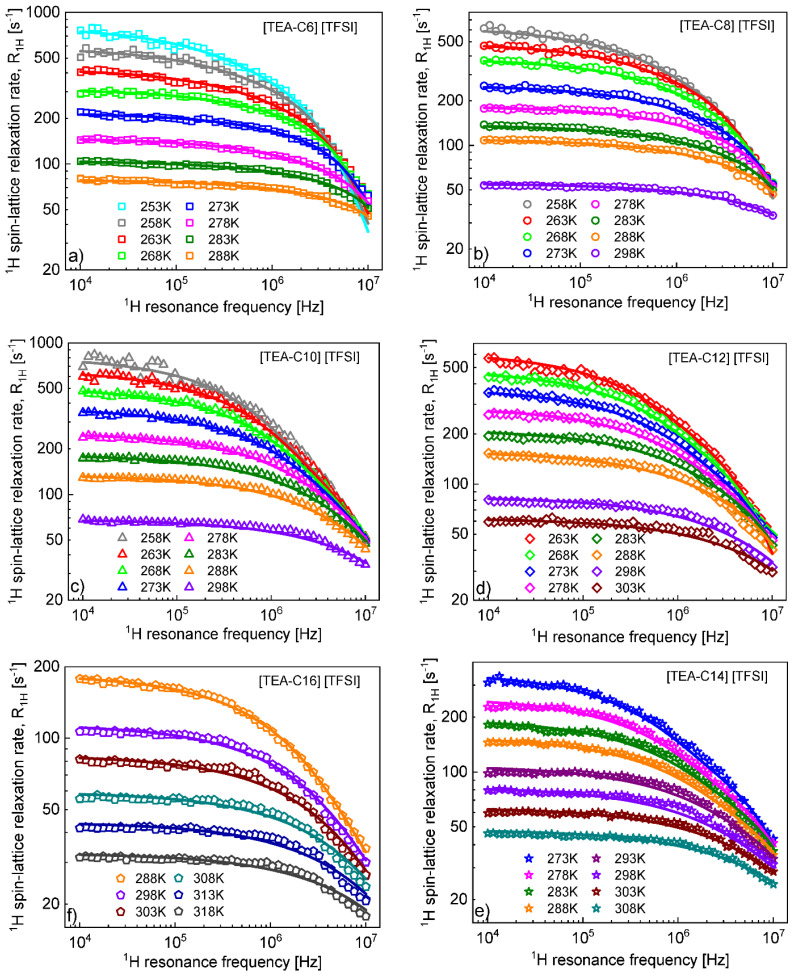
^1^H spin-lattice relaxation data for [TEA-C6] [TFSI] (**a**), [TEA-C8] [TFSI] (**b**), [TEA-C10][TFSI] (**c**), [TEA-C12] [TFSI] (**d**), [TEA-C14] [TFSI] (**e**), and [TEA-C16] [TFSI] (**f**). Solid lines—fits in terms of the model described in Section 3. The experimental uncertainty does not exceed 7%.

^1^H spin-lattice relaxation data obtained for the series of liquids: [TEA-C6] [TFSI], [TEA-C8] [TFSI], [TEA-C10] [TFSI] [TEA-C12] [TFSI], and [TEA-C14] [TFSI] are shown in Figure 1a–e.

The data were analyzed in terms of Equation (1) (with the relaxation rates $R_{1,H}^{inter}\left(\omega \right)$ and $R_{1,H}^{intra}\left(\omega \right)$ given by Equations (2) and (3), respectively) with four adjustable parameters: $C_{DD}^{HH}$, $\tau_{rot}^{C}$, $D_{trans}^{CC}$, and $d_{CC}$; the dipolar relaxation constant, $C_{DD}^{HH}$, and the cation-cation distance of closest approach, $d_{CC}$, were kept temperature independent. The number of ^1^H nuclei (hydrogen atoms) per unit volume was obtained from the relationship: $N_{H}=\frac{n_{H}N_{A}\varrho }{M}$, where $n_{H}$ denotes the number of hydrogen atoms per cation, $N_{A}$ is the Avogadro number, ϱ denotes density of the ionic liquid, while M is its molecular mass. The molecular mass and the density of the liquids are given in Table 1. Consequently, the $N_{H}$ values yield: 4.66 × 10^28^/m^3^ ([TEA-C6] [TFSI]), 4.87 × 10^28^/m^3^ ([TEA-C8] [TFSI]) 5.02 × 10^28^/m^3^ ([TEA-C10] [TFSI]), 5.12 × 10^28^/m^3^ ([TEA-C12] [TFSI]), 5.31 × 10^28^/m^3^ ([TEA-C14] [TFSI]) and 5.38 × 10^28^/m^3^ ([TEA-C16] [TFSI]). The obtained values are included in Table 2. 

$C_{DD}^{HH}$$=8.79\;\times \;108^{\;}{\mathrm{Hz}}^{2},\;d_{CC}$$C_{DD}^{HH}$$=7.77\;\times \;108^{\;}{\mathrm{Hz}}^{2},\;d_{CC}$$C_{DD}^{HH}$$=7.65\;\times \;108^{\;}{\mathrm{Hz}}^{2},\;d_{CC}$$C_{DD}^{HH}$$=7.50\;\times \;108^{\;}{\mathrm{Hz}}^{2},\;d_{CC}$$C_{DD}^{HH}$$=6.50\;\times \;109^{\;}{\mathrm{Hz}}^{2},\;d_{CC}$The diffusion coefficients can also be estimated by means of Equation (4) from the low frequency slopes of the relaxation rates $R_{1H}$ plotted versus $\sqrt{\omega }$. The corresponding figures are shown in Appendix A for [TEA-C6] [TFSI] and [TEA-C8] [TFSI] with the linearity ranges at low frequencies indicated. The corresponding figures for [TEA-C10] [TFSI], [TEA-C12] [TFSI], [TEA-C14] [TFSI], and [TEA-C16] [TFSI] are included into Supplementary Materials. The relative cation-cation translation diffusion coefficients, $D_{trans}^{CC}$, determined from the slopes of the linear dependencies, are collected in Table 2 (the description “slope” indicates the way in which the values were obtained).

**Figure 2:**
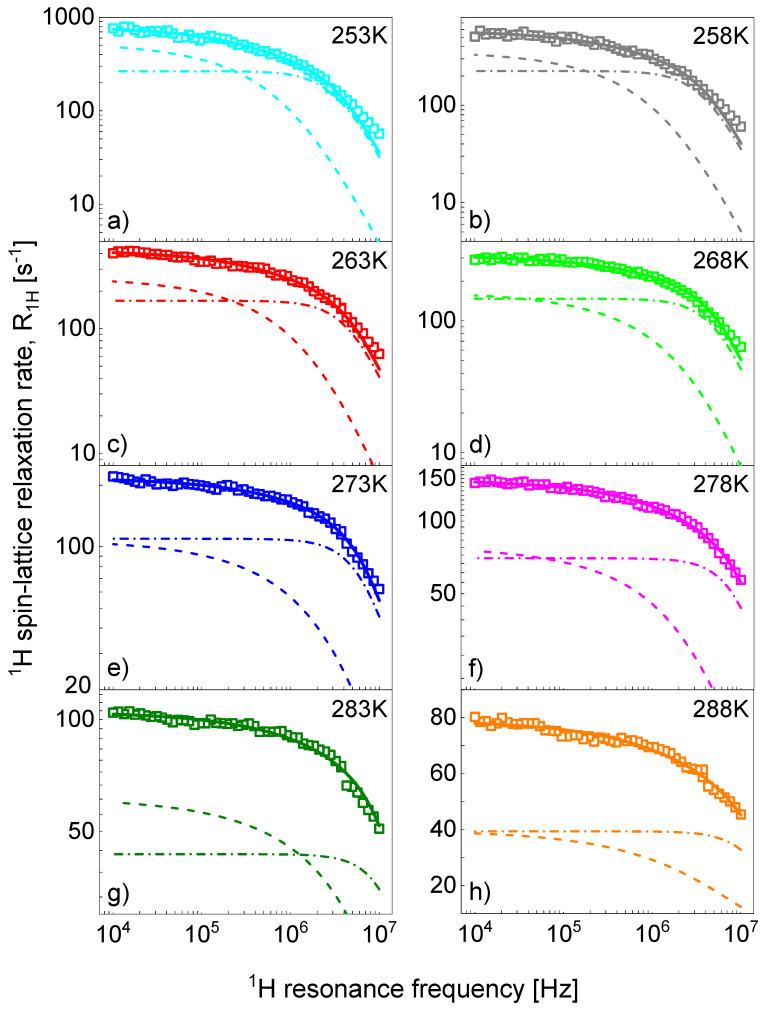
^1^H spin-lattice relaxation data for [TEA-C6] [TFSI] in temperatures 253-288K, (**a**–**h**), respectively. Solid lines—fits in terms of the model described in Section 3 decomposed into the inter-ionic, $R_{1,H}^{inter}\left(\omega \right)$, and intra-ionic, $R_{1,H}^{intra}\left(\omega \right)$; relaxation contributions are represented as dashed and dashed-dotted lines, respectively.

Figure 2 shows a decomposition of the ^1^H spin-lattice relaxation rates for [TEA-C6][TFSI] into the relaxation contribution associated with translation diffusion of the cations, $R_{1,H}^{inter}\left(\omega \right)$, and with their rotational dynamics, $R_{1,H}^{intra}\left(\omega \right)$. Appendix B includes a decomposition of the ^1^H spin-lattice relaxation rates for [TEA-C8][TFSI] as the next example. Analogous decompositions for [TEA-C10] [TFSI], [TEA-C12] [TFSI], [TEA-C14] [TFSI], and [TEA-C16] [TFSI] are shown in Supplementary Materials.

**Figure 3:**
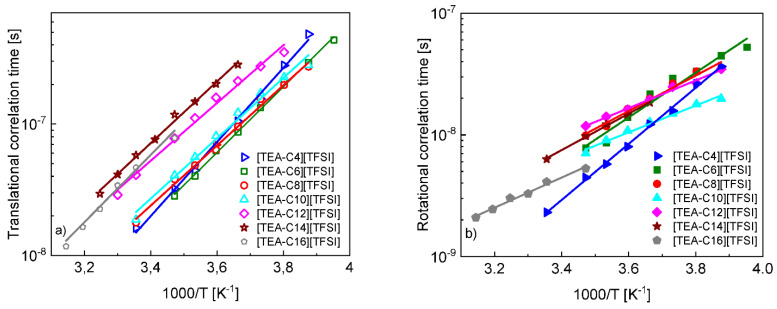
Translational (**a**) and rotational (**b**) correlation times for cations of [TEA-C6] [TFSI], [TEA-C8] [TFSI], [TEA-C10] [TFSI], [TEA-C12] [TFSI], [TEA-C14] [TFSI], and [TEA-C16] [TFSI] versus reciprocal temperature. Solid lines—fits according to the Arrhenius law. The results for [TEA-C4] [TFSI] are taken from [35].

The translational and rotational correlation times of the cations are presented in Figure 3 versus reciprocal temperature. The temperature dependencies follow the Arrhenius law: $\tau =\tau_{0}exp\left(\frac{E_{A}}{kT}\right)$, where τ denotes a correlation time, while $E_{A}$ is an activation energy.

**Figure 4:**
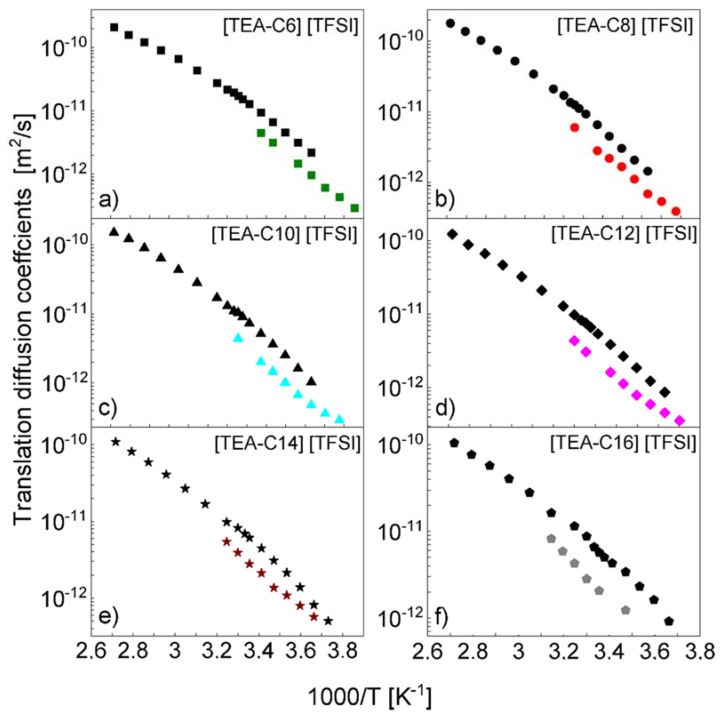
Comparison of translation diffusion coefficients of cations obtained for [TEA-C6] [TFSI] (**a**), [TEA-C8] [TFSI] (**b**), [TEA-C10] [TFSI] (**c**), [TEA-C12] [TFSI] (**d**), [TEA-C14] [TFSI] (**e**), and [TEA-C16] [TFSI] (**f**) by means of NMR relaxometry (color points) and diffusometry (black points); the last values were taken from [37] and multiplied by the factor of two.

Eventually, it is worth comparing the translation diffusion coefficients obtained from the current analysis with those measured by NMR diffusometry. The comparison is shown in Figure 4; the diffusion coefficients from [37] were multiplied by the factor of two to account for the relative translational motion. One should note that although in the temperature range covered by the NMR relaxometry studies, the translation diffusion coefficients follow the Arrhenius law, deviations from the Arrhenius dependence are observed in the broad temperature range encompassing both the diffusometry and the relaxometry results.

The obtained results are discussed in the next section.

## 5. Discussion

The set of ^1^H spin-lattice relaxation data for [TEA-C6] [TFSI], [TEA-C8] [TFSI], [TEA-C10] [TFSI], [TEA-C12] [TFSI], [TEA-C14] [TFSI], and [TEA-C16] [TFSI] gives rise to the translation diffusion coefficients (correlation times) and the rotational correlation times. The first observation that can be made is that the ratio between the correlation times, $\frac{\tau_{trans}}{\tau_{rot}}$, for this series of ionic liquids is much lower compared to that for molecular liquids [15,16,17,18]; one should include to this series also [TEA-C4] [TFSI] [35]. This finding cannot be generalized, as there is a lot of data available for molecular liquids; this kind of result is scarce for ionic liquids. This implies that the $\frac{\tau_{trans}}{\tau_{rot}}$ ratio for this series of ionic liquids, averaged over temperature, is more close to the theoretical value of 9 predicted by the Stokes–Einstein equation for the idealistic case of spherical molecules (although the cations are far from being spherical), while the typical values of molecular liquids are of the order of 30–40 [15,16,17,18]. However, one should note that the ratios of Table 2 considerably depend on temperature, reaching at higher temperatures values even close to 3 ([TEA-C8] [TFSI] at 298 K).

It is of interest to compare the temperature dependencies of the translational and rotational correlation times (Figure 3a,b). The activation energy of the translation dynamics ranges from (23.4 ± 0.6) kJ/(mol × K) for [TEA-C4][TFSI] to (18.3 ± 0.7) kJ/(mol × K) for [TEA-C12] [TFSI] (the other values are: (20.8 ± 0.4) kJ/(mol × K) for [TEA-C6] [TFSI], (19.0 ± 0.6) kJ/(mol × K) for [TEA-C8] [TFSI], (19.0 ± 0.9) kJ/(mol × K) for [TEA-C10] [TFSI], (19.5 ± 0.5) kJ/(mol × K) for [TEA-C14] [TFSI] and (21.3 ± 1.0) kJ/(mol × K) for [TEA-C16] [TFSI]), spanning a narrow range of values (the reciprocal temperature dependencies of the translational correlation times for the different liquids are almost parallel). In contrast to that, the activation energies for the rotational dynamics span the range of (19.2 ± 0.4) kJ/(mol × K) for [TEA-C4] [TFSI] to (9.1 ± 0.5) kJ/(mol × K) for [TEA-C10] [TFSI] (the other values are: (15.3 ± 1.2) kJ/(mol × K) for [TEA-C6] [TFSI], (12.1 ± 0.8) kJ/(mol × K) for [TEA-C8] [TFSI], (9.4 ± 0.4) kJ/(mol × K) for [TEA-C12][TFSI], (12.5 ± 0.4) kJ/(mol × K) for [TEA-C14] [TFSI] and (10.3 ± 0.6) kJ/(mol × K) for [TEA-C16] [TFSI]).The range of the activation energies is considerably broader with no systematic dependence of the alkyl chain length. The same one can say about the rotational correlation times—the values do not vary monotonically with the increasing alkyl chain length. The ratio between the activation energies of the translational and rotational motion varies between 1.2 for [TEA-C4] [TFSI] and 2.1 for [TEA-C10] [TFSI].

As already pointed out, the translation diffusion coefficients obtained by means of NMR relaxometry describe the relative, cation-cation translation movement, while NMR diffusometry allows to determine the self-diffusion coefficient of the cation. For uncorrelated dynamics, the relative translation diffusion should be twice larger than the self-diffusion one. This relationship has been demonstrated for a variety of molecular liquids [23]. Our results indicate that this does not apply to ionic liquids; in all cases (including [TEA-C4] [TFSI] [35]), the relative diffusion coefficients are lower than the self-diffusion ones multiplied by two, as shown in Figure 4. This finding indicates a correlated translation movement of the cations.

Eventually, from the point of view of the methodology, one should stress that although we strongly recommend to perform thorough analysis of relaxation data for ionic liquids to fully profit from the potential of NMR relaxometry to reveal the translational and rotational dynamics in a single experiment, translation diffusion coefficients can be estimated from the low-frequency slope with accuracy (Table 2) that is sufficient for application purposes.

## 6. Conclusions

A large set of ^1^H spin-lattice relaxation data for a series of ionic liquids [TEA-C6] [TFSI], [TEA-C8] [TFSI], [TEA-C10] [TFSI], [TEA-C12] [TFSI], [TEA-C14] [TFSI], and [TEA-C16] [TFSI] (the structure of the cation systematically changes) were thoroughly analyzed in terms of a relaxation model including inter-ionic (cation-cation) and intra-ionic relaxation contributions associated with relative translation movement of the cations and their rotational dynamics, respectively. The obtained results allowed to enquire into the relationship between the translational and rotational dynamics of this series of ionic liquids, in comparison to corresponding characteristic features of molecular liquids. It was shown that the ratio between the translational and rotational correlation times for the ionic liquids is lower compared to values obtained for molecular liquids and changes with temperature more significantly. Both the translational and the rotational dynamics turned out to follow the Arrhenius law; however, while the activation energies for the translation dynamics are similar for all liquids (they range from 18.3 kJ/(mol × K) for [TEA-C12] [TFSI] to 23.4 kJ/(mol × K) for [TEA-C4] [TFSI]), the activation energies for the rotational dynamics vary in a rather broad range (from 9.1 kJ/(mol × K) for [TEA-C4] [TFSI] to 19.2 kJ/(mol × K) for [TEA-C4] [TFSI]). The values of the rotational activation energy do not change monotonically with the alkyl chain length. The rotational activation energies are lower than the translational ones; the ratio between the activation energies of the translational and rotational motions reaches the lowest value of 1.2 for [TEA-C4] [TFSI] and the highest value of 2.1 for [TEA-C10] [TFSI]. Moreover, it was shown that the relative translation diffusion coefficients for all liquids are lower than twice the corresponding self-diffusion coefficients, indicating correlation effects in the translation movement.

The presented studies are a step towards revealing generic features of ionic liquids with respect to their translational and rotational dynamics. It was, for instance, shown that for the whole series of ionic liquids (independently of the chain length), the ratio between the translational and rotational correlation times is much closer to the predictions of the Stokes–Einstein equation than has been observed for molecular liquids.

NMR relaxometry is a unique method enabling to probe translational and rotational dynamics in a single experiment, and this potential has been exploited for molecular liquids, but not for ionic systems, so far.

## Figures and Tables

**Table 1:** Basic properties of ionic liquids.

Name; Abbreviation	Chemical Formula	Molecular Mass [g/mol]	Density [g/cm^3^]	$T_{cryst}\;[K]$
Triethylhexylammonium bis(trifluoromethanesulfonyl)imide; [TEA-C6] [TFSI]	C_14_H_28_F_6_N_2_O_4_S_2_	466.50	1.29	228.18
Triethyloctylammonium bis(trifluoromethanesulfonyl)imide; [TEA-C8] [TFSI]	C_16_H_32_F_6_N_2_O_4_S_2_	494.56	1.25	231.83
Decyltriethylammonium bis(trifluoromethanesulfonyl)imide; [TEA-C10] [TFSI]	C_18_H_36_F_6_N_2_O_4_S_2_	522.61	1.21	-
Dodecyltriethylammonium bis(trifluoromethanesulfonyl)imide; [TEA-C12] [TFSI]	C_20_H_40_F_6_N_2_O_4_S_2_	550.66	1.17	-
Triethyltetradecylammonium bis(trifluoromethanesulfonyl)imide; [TEA-C14] [TFSI]	C_22_H_44_F_6_N_2_O_4_S_2_	578.72	1.16	240.03
Hexadecyltriethylammonium bis(trifluoromethanesulfonyl)imide [TEA-C16] [TFSI]	C_24_H_48_F_6_N_2_O_4_S_2_	606.77	1.13	261.69

**Table 2:** Parameters characterizing the translational and rotational dynamics of TEA-C6, TEA-C8, TEA-C10, TEA-C12, TEA-C14, and TEA-C16 cations. The correlation time $\tau_{trans}^{C}$ was calculated from the relationship:$\;\tau_{trans}^{C}=\frac{d_{CC}^{2}}{D_{trans}^{CC}}$.

Temp. [K]	$D_{trans}^{CC}\;[m^{2}/s]$	$D_{trans}^{CC}\;[m^{2}/s]\;(\mathrm{Slope})$	$\tau_{rot}^{C}\;[s]$	Rel. Error [%]	$\tau_{trans}^{C}\;[s]$	$\tau_{trans}^{C}$ $/\tau_{rot}^{C}$
**TEA-C6**					$C_{DD}^{HH}$ **= 9.96 × 10^8^ Hz^2^,** $d_{CC}$ ** = 3.57 Å**	
253	2.92 × 10^−13^	4.16 × 10^−13^	5.26 × 10^−8^	10.4	4.36 × 10^−7^	8.3
258	4.35 × 10^−13^	5.76 × 10^−13^	4.46 × 10^−8^	8.9	2.93 × 10^−7^	6.6
263	6.11 × 10^−13^	7.54 × 10^−13^	3.32 × 10^−8^	8.4	2.09 × 10^−7^	6.3
268	9.55 × 10^−13^	1.23 × 10^−12^	2.92 × 10^−8^	6.5	1.33 × 10^−7^	4.6
273	1.47 × 10^−12^	1.85 × 10^−12^	2.17 × 10^−8^	4.4	8.67 × 10^−8^	4.0
278	2.04 × 10^−12^	2.62 × 10^−12^	1.39 × 10^−8^	2.0	6.25 × 10^−8^	4.5
283	3.17 × 10^−12^	4.06 × 10^−12^	8.62 × 10^−9^	3.9	4.02 × 10^−8^	4.7
288	4.50 × 10^−12^	5.22 × 10^−12^	7.80 × 10^−9^	3.3	2.83 × 10^−8^	3.6
**TEA-C8**					$C_{DD}^{HH}$**= 8.79 × 10^8^ Hz^2^,**$d_{CC}$ **=3.26 Å**	
258	3.89 × 10^−13^	4.74 × 10^−13^	3.50 × 10^−8^	14.6	2.73 × 10^−7^	7.8
263	5.36 × 10^−13^	7.00 × 10^−13^	3.31 × 10^−8^	12.2	1.98 × 10^−7^	6.0
268	6.87 × 10^−13^	9.02 × 10^−13^	2.64 × 10^−8^	8.3	1.55 × 10^−7^	5.9
273	1.11 × 10^−12^	1.54 × 10^−12^	1.98 × 10^−8^	8.0	9.57 × 10^−8^	4.8
278	1.67 × 10^−12^	2.28 × 10^−12^	1.63 × 10^−8^	11.5	6.36 × 10^−8^	3.9
283	2.19 × 10^−12^	2.76 × 10^−12^	1.18 × 10^−8^	3.7	4.85 × 10^−8^	4.1
288	2.80 × 10^−12^	3.50 × 10^−12^	9.87 × 10^−9^	3.6	3.80 × 10^−8^	3.9
298	5.97 × 10^−12^	7.71 × 10^−12^	5.42 × 10^−9^	4.7	1.78 × 10^−8^	3.3
**TEA-C10**					$C_{DD}^{HH}$**= 7.77 × 10^8^ Hz^2^,**$d_{CC}$ **=2.86 Å**	
258	2.91 × 10^−13^	3.20 × 10^−13^	1.98 × 10^−8^	16.2	2.81 × 10^−7^	14.2
263	3.61 × 10^−13^	4.70 × 10^−13^	1.79 × 10^−8^	13.9	2.27 × 10^−7^	12.7
268	4.78 × 10^−13^	6.34 × 10^−13^	1.49 × 10^−8^	6.2	1.71 × 10^−7^	11.5
273	6.70 × 10^−13^	9.20 × 10^−13^	1.27 × 10^−8^	5.3	1.22 × 10^−7^	9.6
278	1.01 × 10^−12^	1.44 × 10^−12^	1.08 × 10^−8^	5.6	8.10 × 10^−8^	7.5
283	1.46 × 10^−12^	2.00 × 10^−12^	9.02 × 10^−9^	8.3	5.60 × 10^−8^	6.2
288	2.01 × 10^−12^	2.69 × 10^−12^	7.06 × 10^−9^	7.7	4.07 × 10^−8^	5.8
298	4.37 × 10^−12^	5.49 × 10^−12^	4.76 × 10^−9^	7.9	1.87 × 10^−8^	3.9
**TEA-C12**					$C_{DD}^{HH}$**= 7.65 × 10^8^ Hz^2^,**$d_{CC}$ **=3.54 Å**	
263	3.56 × 10^−13^	5.34 × 10^−13^	3.14× 10^−8^	13.3	3.52× 10^−7^	11.2
268	4.55 × 10^−13^	6.58 × 10^−13^	2.48× 10^−8^	9.2	2.75× 10^−7^	11.1
273	5.91 × 10^−13^	8.10 × 10^−13^	1.94× 10^−8^	5.3	2.12× 10^−7^	10.9
278	7.87 × 10^−13^	1.13 × 10^−12^	1.61× 10^−8^	10.1	1.59× 10^−7^	9.9
283	1.13 × 10^−12^	1.62 × 10^−12^	1.41× 10^−8^	16.5	1.11× 10^−7^	7.8
288	1.61 × 10^−12^	2.36× 10^−12^	1.18× 10^−8^	11.4	7.78× 10^−8^	6.6
298	3.06 × 10^−12^	3.73× 10^−12^	6.51× 10^−9^	14.2	4.10× 10^−8^	6.3
303	4.34 × 10^−12^	4.33× 10^−12^	5.38× 10^−9^	15.7	2.89× 10^−8^	5.4
**TEA-C14**					$C_{DD}^{HH}$**= 7.50 × 10^8^ Hz^2^,**$d_{CC}$ **=4.02 Å**	
273	5.72 × 10^−13^	8.13 × 10^−13^	1.84 × 10^−8^	11.6	2.83 × 10^−7^	15.4
278	8.01 × 10^−13^	1.16 × 10^−12^	1.45 × 10^−8^	15.1	2.02 × 10^−7^	13.9
283	1.09 × 10^−12^	1.66 × 10^−12^	1.22 × 10^−8^	14.7	1.48 × 10^−7^	12.2
288	1.37 × 10^−12^	2.00 × 10^−12^	9.96 × 10^−9^	10.8	1.18 × 10^−7^	11.8
293	2.12 × 10^−12^	3.21 × 10^−12^	8.32 × 10^−9^	18.9	7.62 × 10^−8^	9.2
298	2.79 × 10^−12^	3.96 × 10^−12^	6.41 × 10^−9^	18.1	5.79 × 10^−8^	9.0
303	3.90 × 10^−12^	5.37 × 10^−12^	5.76 × 10^−9^	14.6	4.14 × 10^−8^	7.2
308	5.43 × 10^−12^	7.42 × 10^−12^	4.72 × 10^−9^	13.4	2.95 × 10^−8^	5.4
**TEA-C16**					$C_{DD}^{HH}$**= 6.50 × 10^8^ Hz^2^,**$d_{CC}$ **= 3.17Å**	
288	1.23 × 10^−12^	1.57 × 10^−12^	5.29 × 10^−9^	2.6	8.17 × 10^−8^	15.4
298	2.07 × 10^−12^	3.31 × 10^−12^	4.11 × 10^−9^	8.2	4.85 × 10^−8^	11.8
303	2.83 × 10^−12^	5.11 × 10^−12^	3.28 × 10^−9^	12.7	3.55 × 10^−8^	10.8
308	4.27 × 10^−12^	7.02 × 10^−12^	3.02 × 10^−9^	11.1	2.35 × 10^−8^	7.8
313	5.86 × 10^−12^	8.57 × 10^−12^	2.45 × 10^−9^	11.0	1.71 × 10^−8^	7.0
318	8.21 × 10^−12^	1.36 × 10^−11^	2.09 × 10^−9^	9.6	1.22 × 10^−8^	5.8

**Figure A1:**
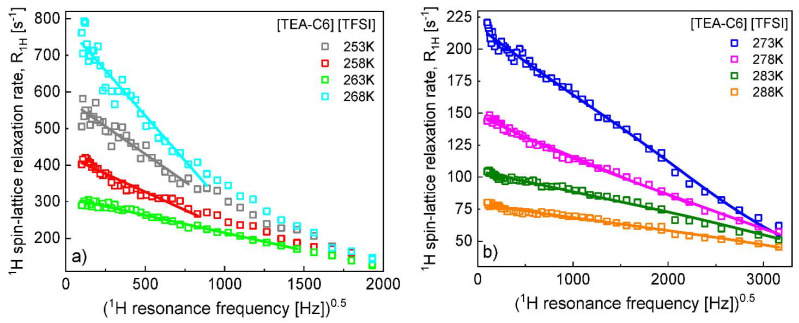
(**a**,**b**) ^1^H spin-lattice relaxation data for [TEA-C6] [TFSI] versus squared root of resonance frequency; solid lines represent linear fits in the low frequency range.

**Figure A2:**
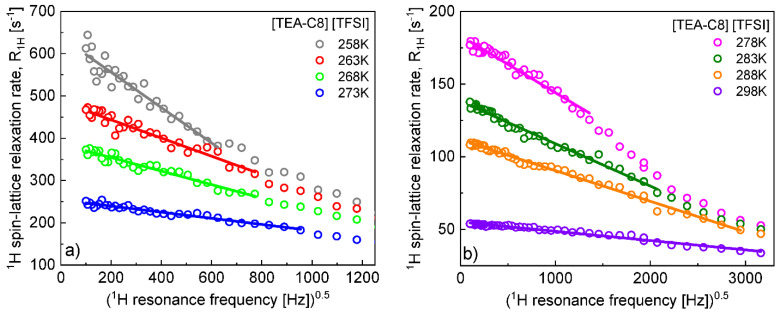
(**a**,**b**) ^1^H spin-lattice relaxation data for [TEA-C8] [TFSI] versus squared root of resonance frequency; solid lines represent linear fits in the low frequency range.

**Figure A3:**
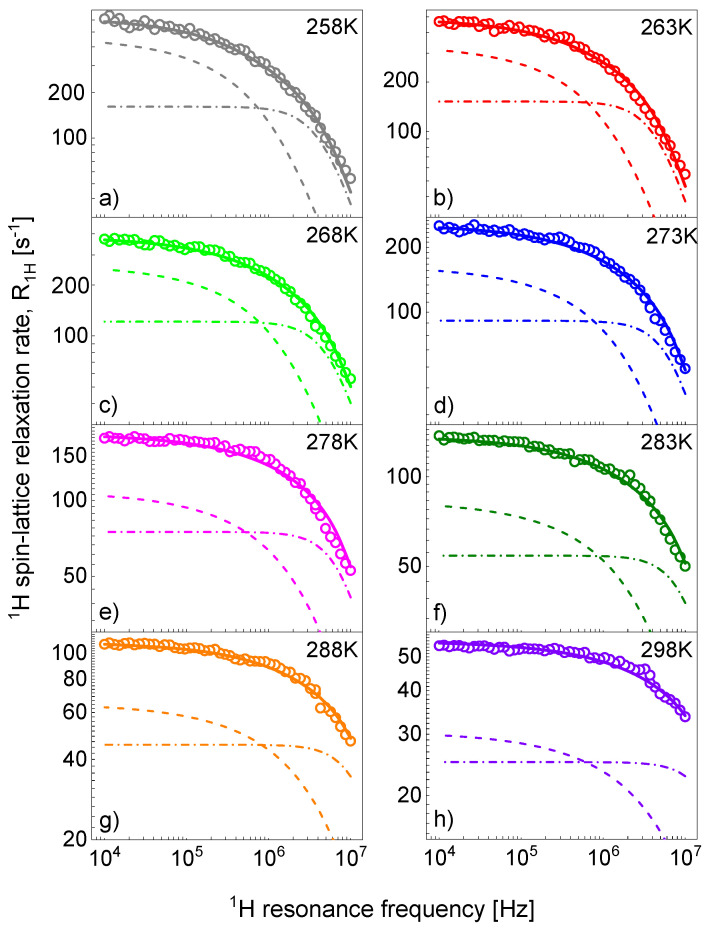
^1^H spin-lattice relaxation data for [TEA-C8] [TFSI] in temperatures 258-298K, (**a**–**h**), repsectively. Solid lines—fits in terms of the model described in Section 3 decomposed into the inter-ionic, $R_{1,H}^{inter}\left(\omega \right)$, and intra-ionic, $R_{1,H}^{intra}\left(\omega \right)$; relaxation contributions are represented as dashed and dashed-dotted lines, respectively.

## Data Availability

Data are available from the corresponding Author (D.K.)

## Supplementary Materials

The following supporting information can be downloaded at: https://www.mdpi.com/article/10.3390/ijms23031688/s1.

## Appendix A

Linear dependencies of ^1^H spin-lattice relaxation rates on squared root of the resonance frequency.

## Appendix B

Decomposition of the overall relaxation rates into the inter-ionic and intra-ionic relaxation contributions.
