# Supplementary material for: Relationship between Translational and Rotational Dynamics of Alkyltriethylammonium-Based Ionic Liquids
Source: Int J Mol Sci. 2022 Feb 1;23(3):1688. doi: 10.3390/ijms23031688 (PMC8836145; doi:10.3390/ijms23031688)
Supplement: Supplementary file 1 [file ijms-23-01688-s001.zip › ijms-1506265-supplementary.pdf]

Supplementary Materials

Examples of ^{1}H magnetization curves (magnetization versus time). Solid lines indicate single exponential fits.

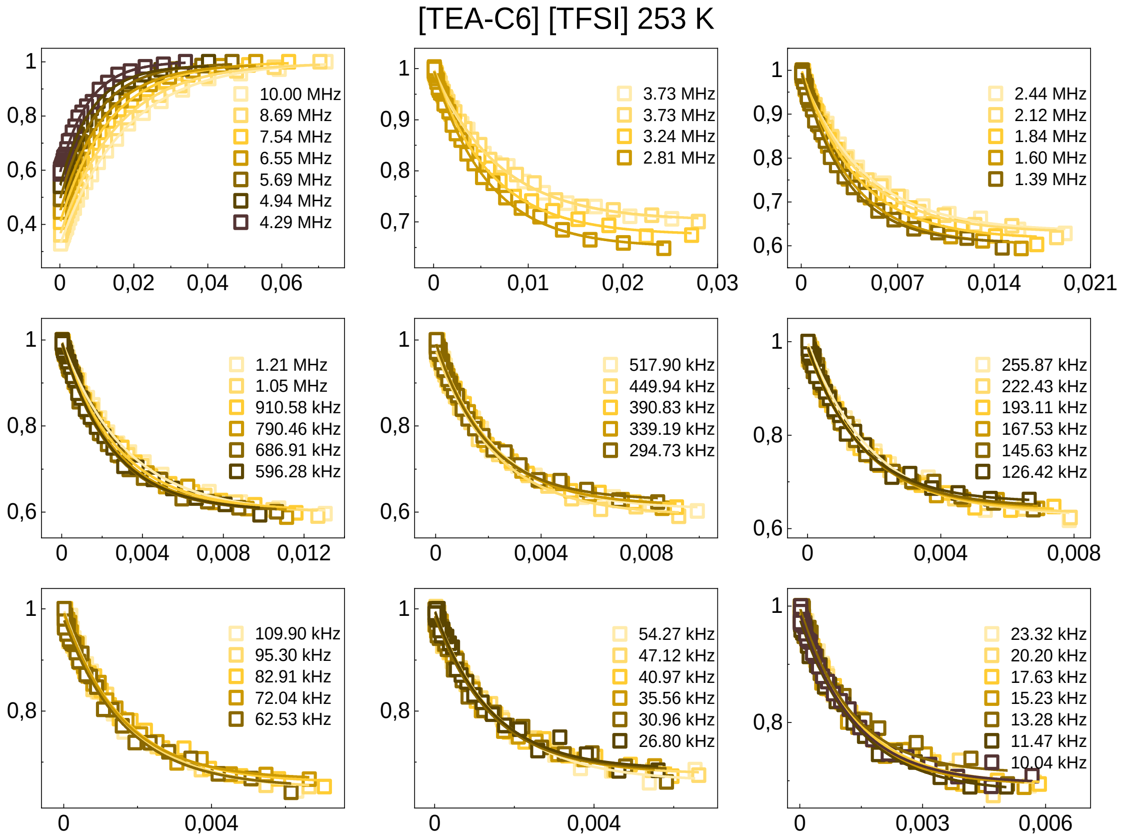

Figure S1. ^{1}H magnetization curves for [TEA-C6] [TFSI] at 253K.

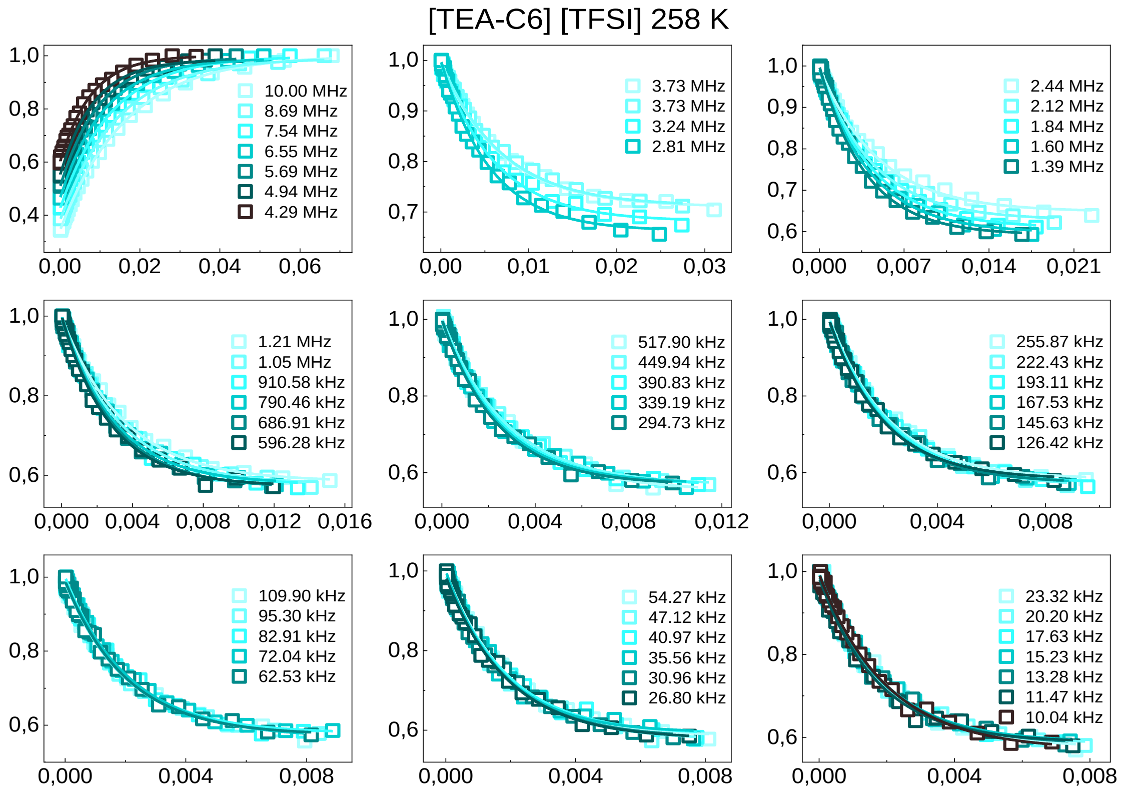

Figure S2. ^{1}H magnetization curves for [TEA-C6] [TFSI] at 258K.

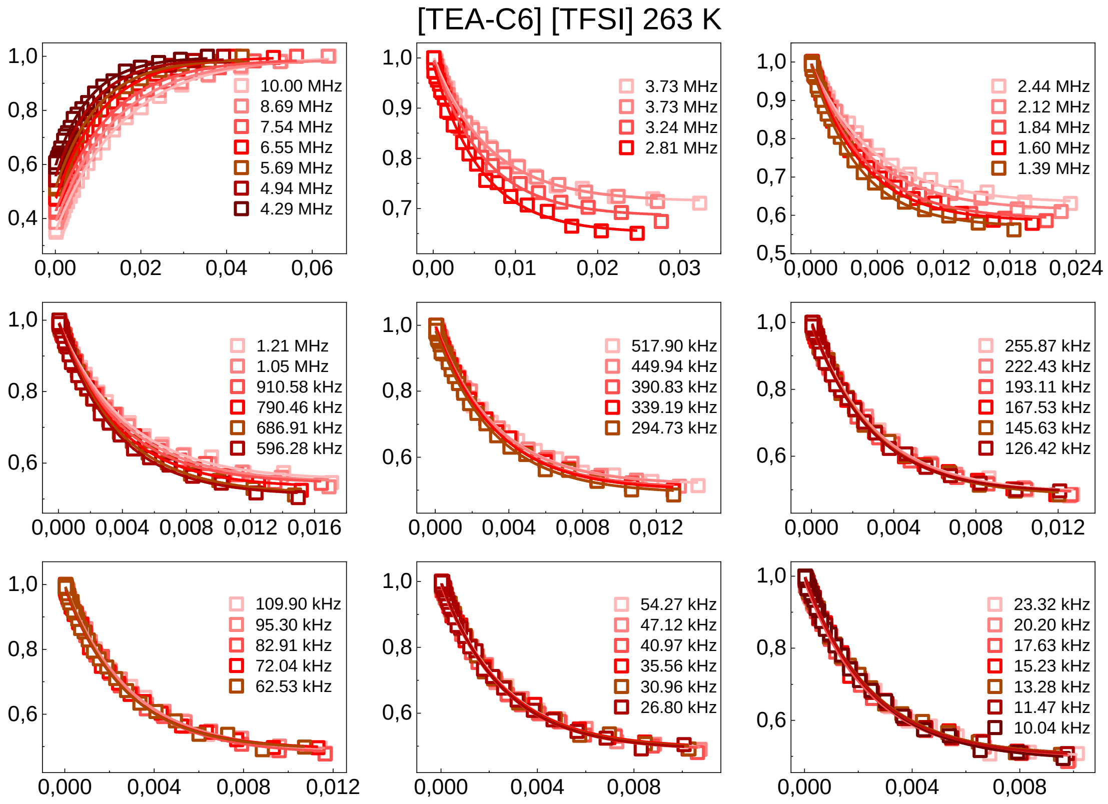

Figure S3. [1]H magnetization curves for [TEA-C6] [TFSI] at 263K.

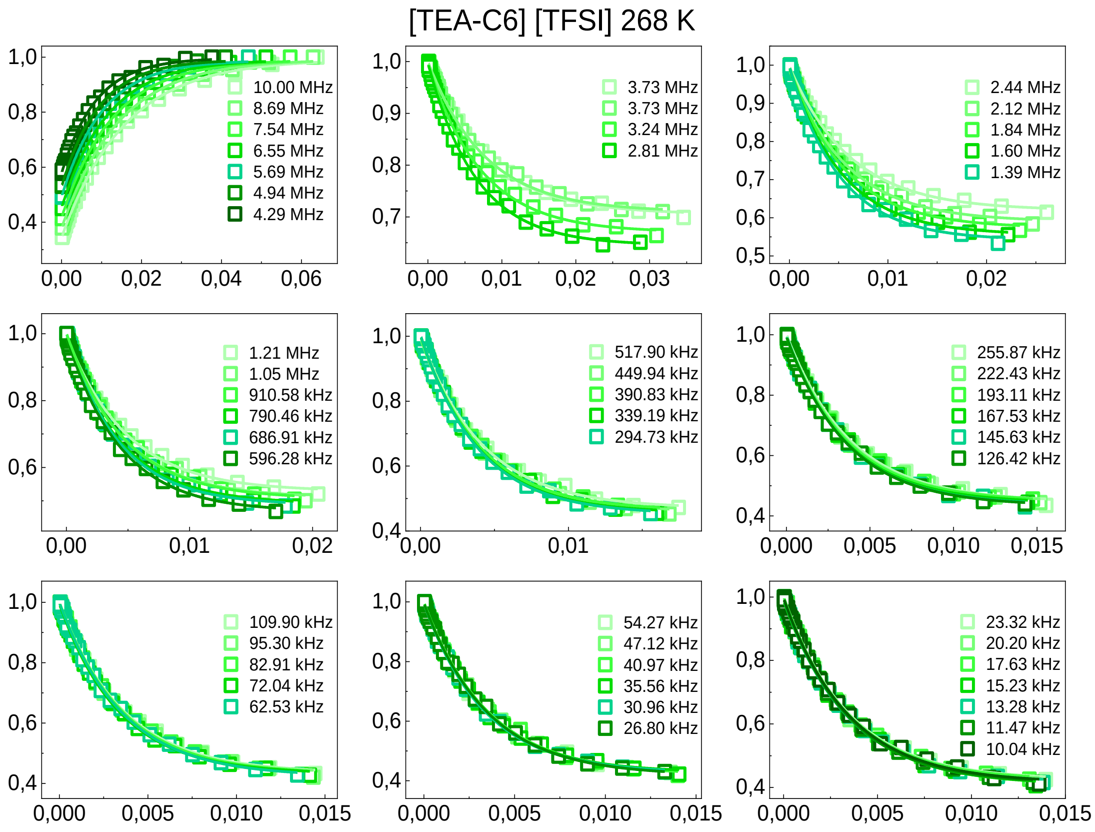

Figure S4. [1]H magnetization curves for [TEA-C6] [TFSI] at 263K.

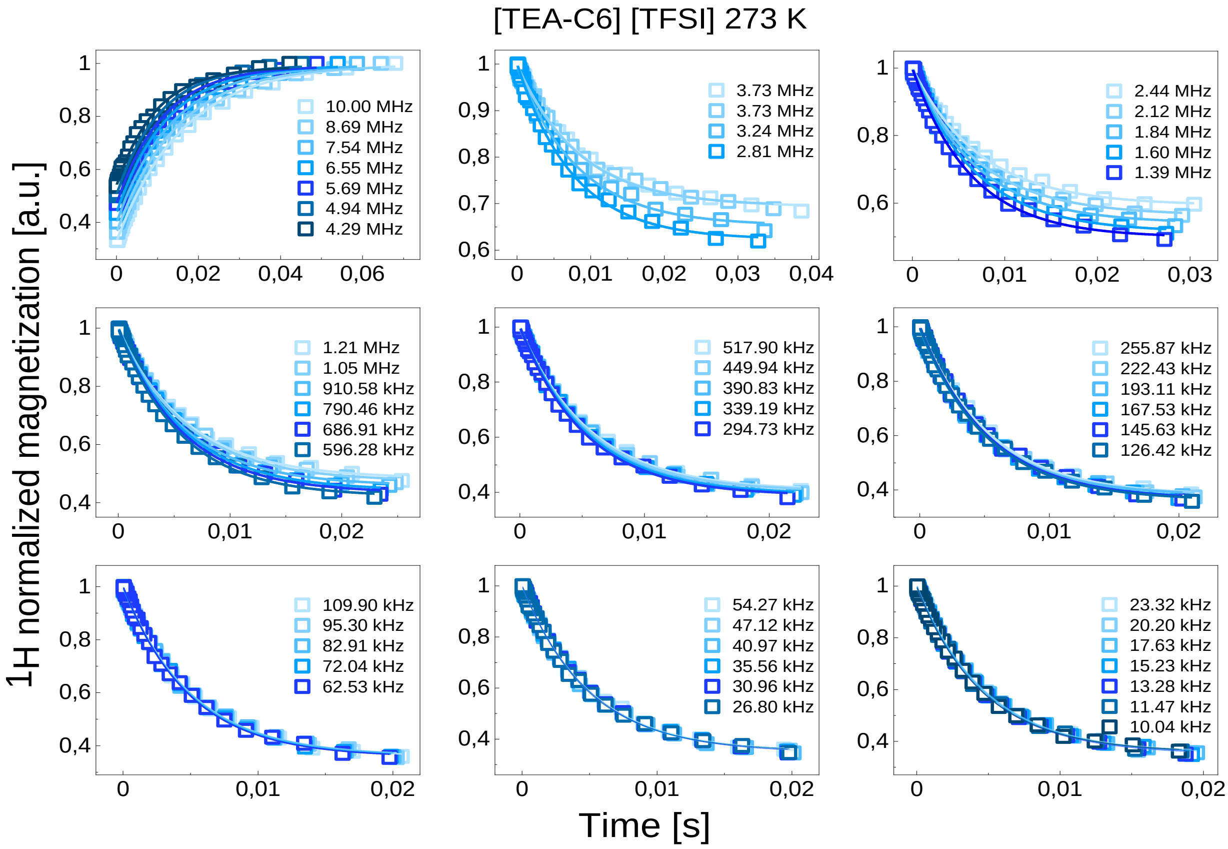

Figure S5. ^{1}H magnetization curves for [TEA-C6] [TFSI] at 273K.

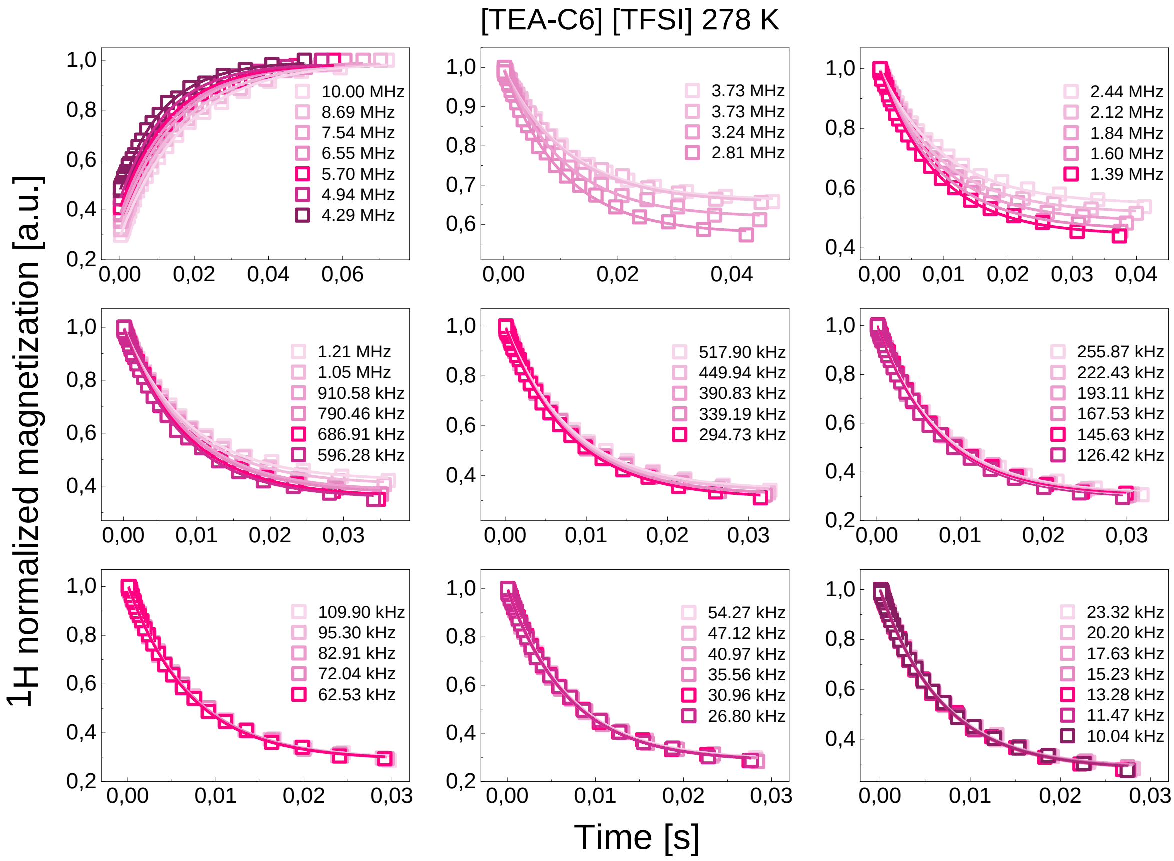

Figure S6. ^{1}H magnetization curves for [TEA-C6] [TFSI] at 278K.

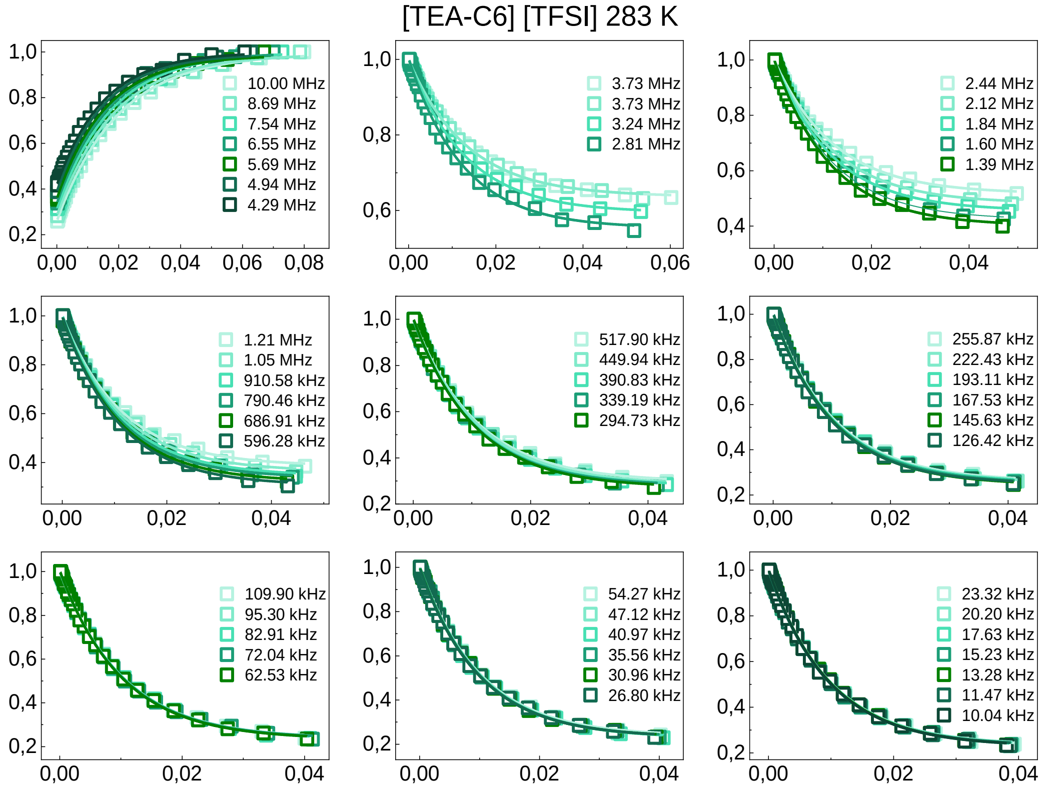

Figure S7. [1]H magnetization curves for [TEA-C6] [TFSI] at 283K.

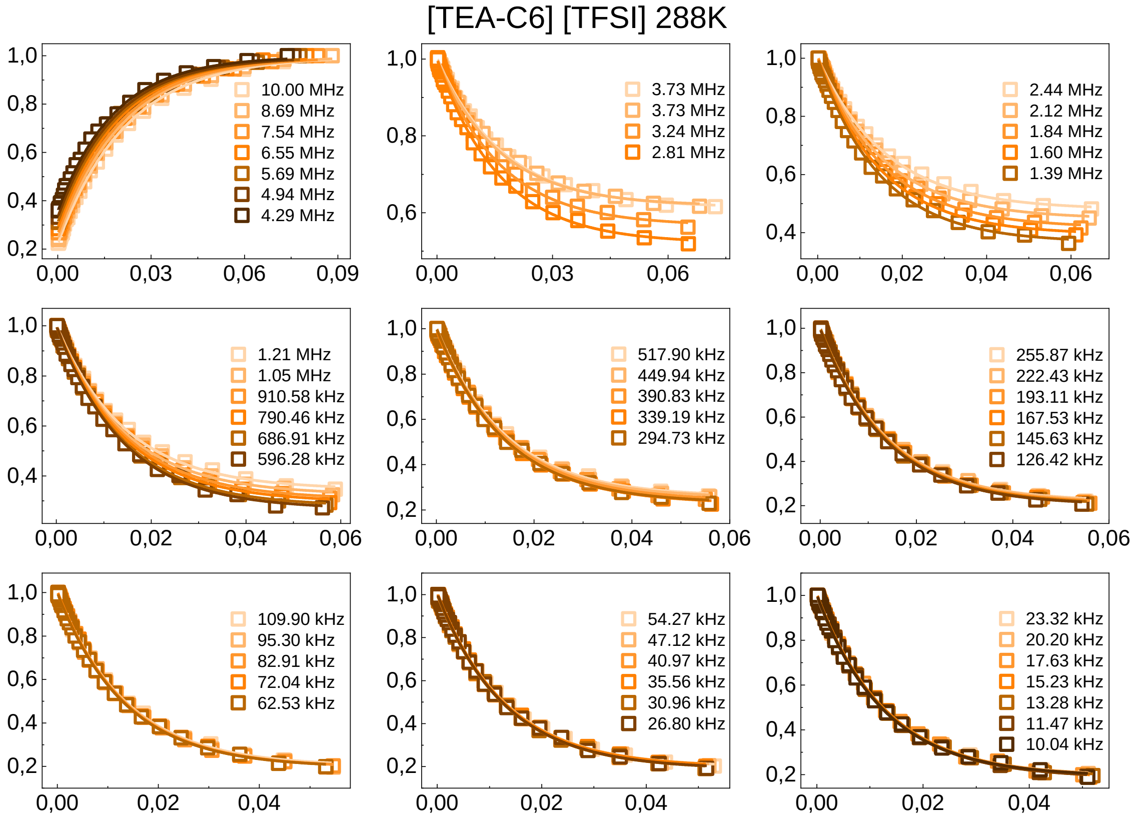

Figure S8. ^{1}H magnetization curves for [TEA-C6] [TFSI] at 288K.

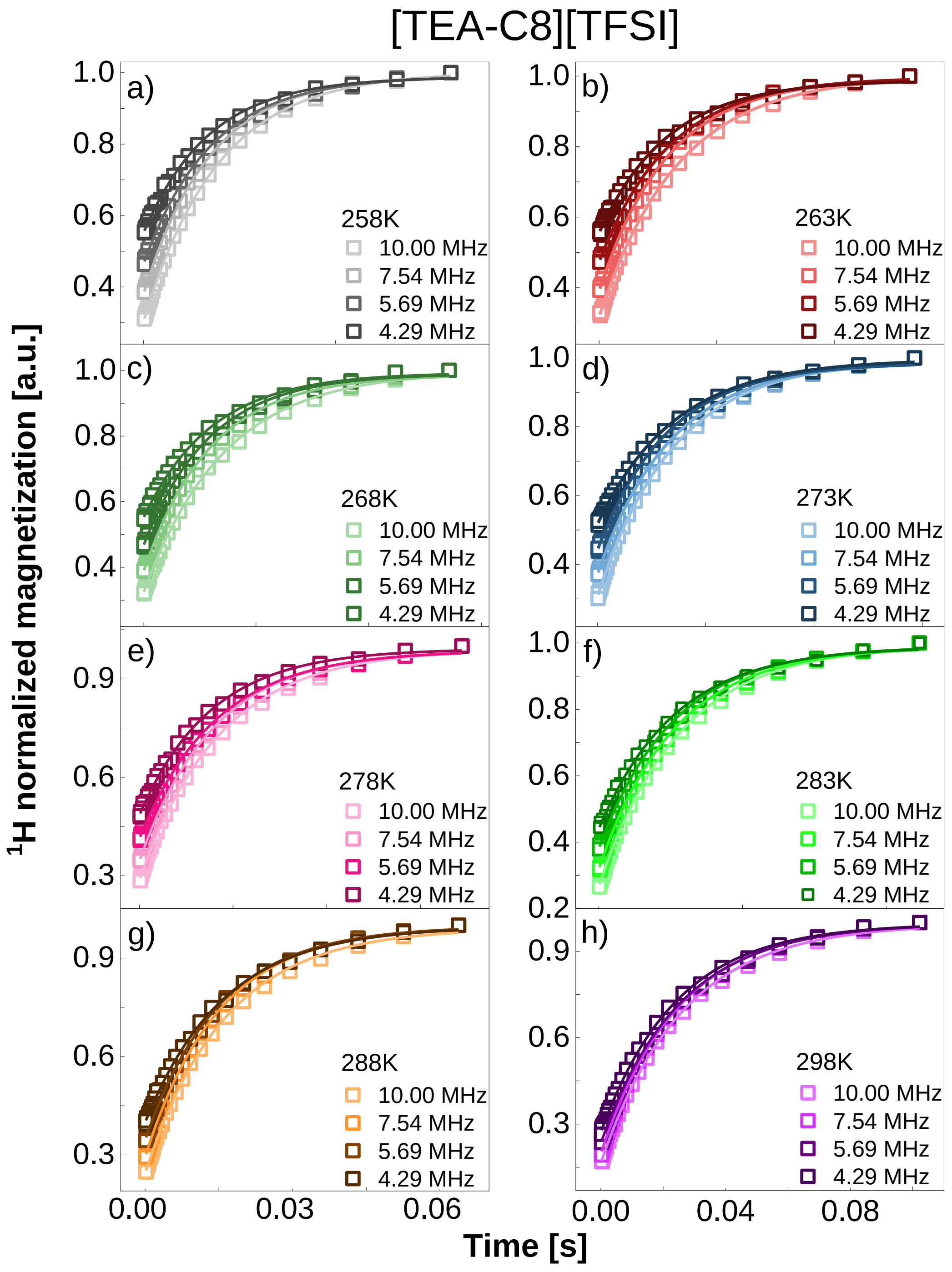

Figure S9. ^{1}H magnetization curves for [TEA-C8] [TFSI].

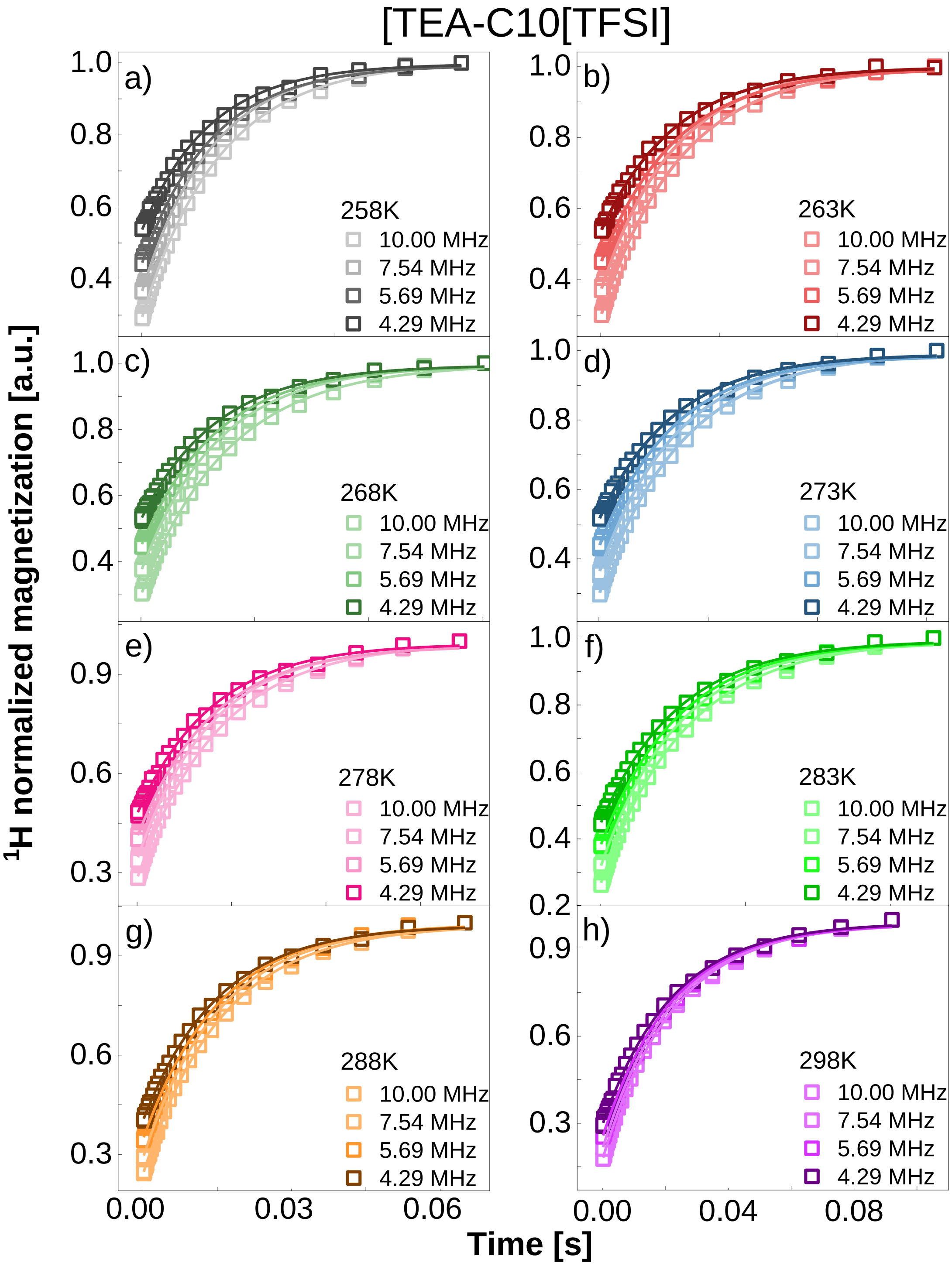

Figure S10. ^{1}H magnetization curves for [TEA-C10] [TFSI].

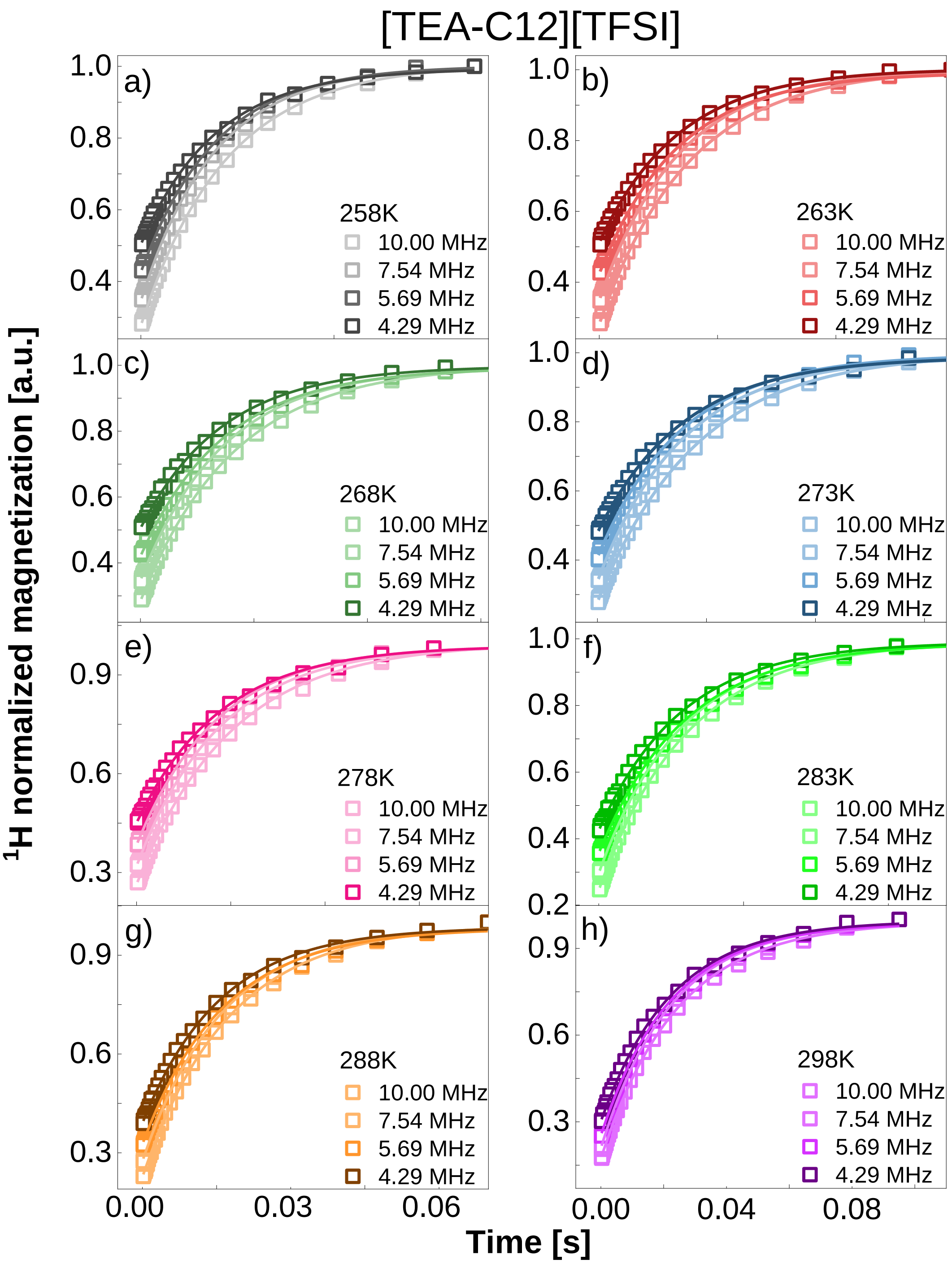

Figure S11. ^{1}H magnetization curves for [TEA-C12] [TFSI].

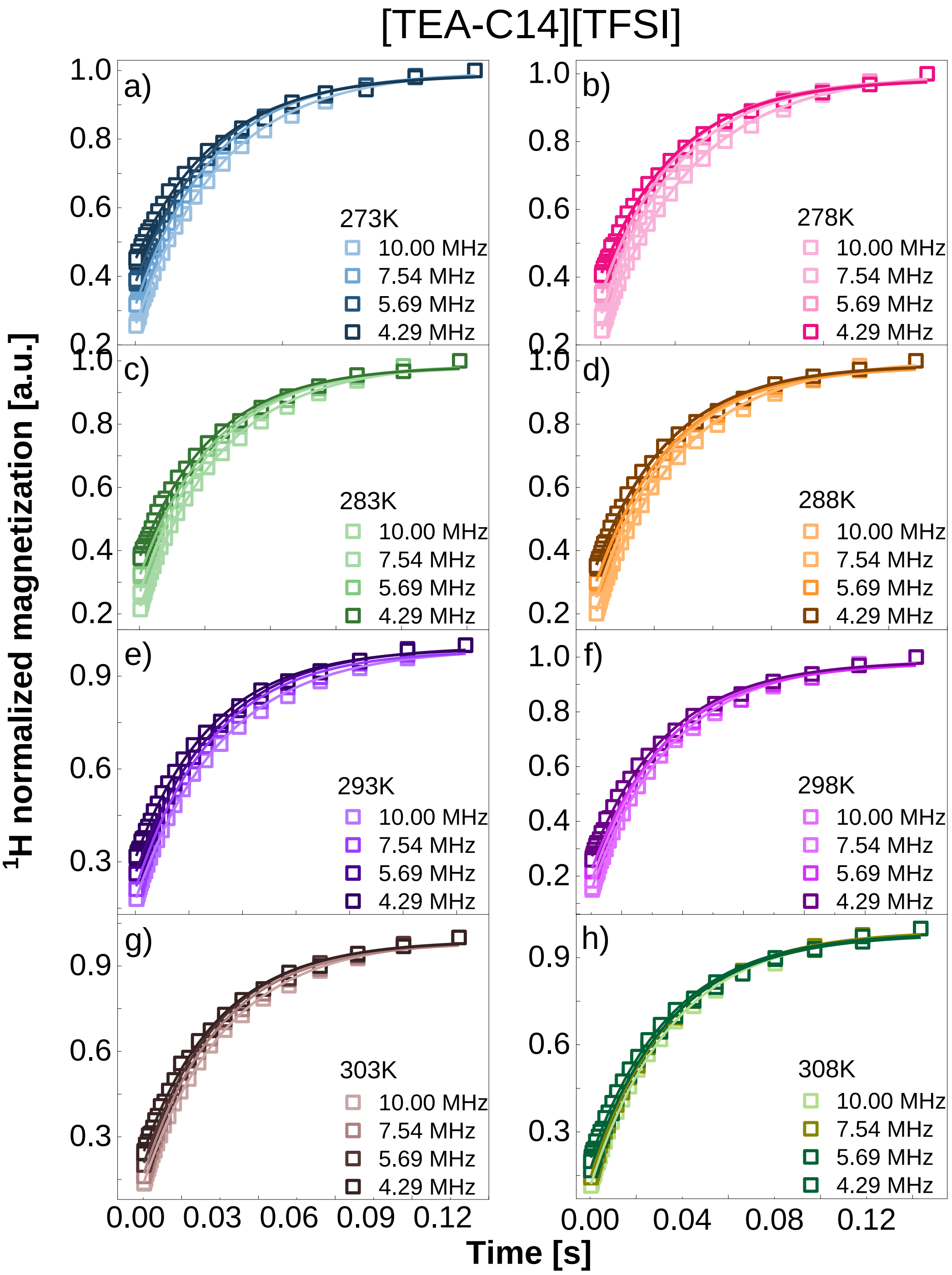

Figure S12. ^{1}H magnetization curves for [TEA-C14] [TFSI].

[1]H spin-lattice relaxation rates versus squared root of the resonance frequency.

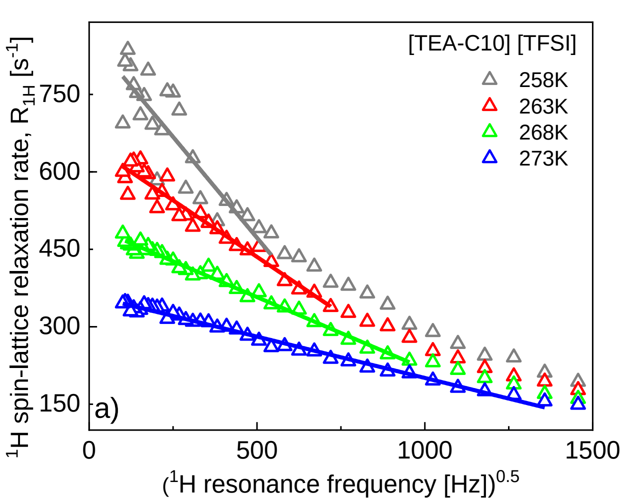

Figure S13a,b. [1]H spin-lattice relaxation data for [TEA-C10] [TFSI] versus squared root of resonance frequency; solid lines represent linear fits in the low frequency range.

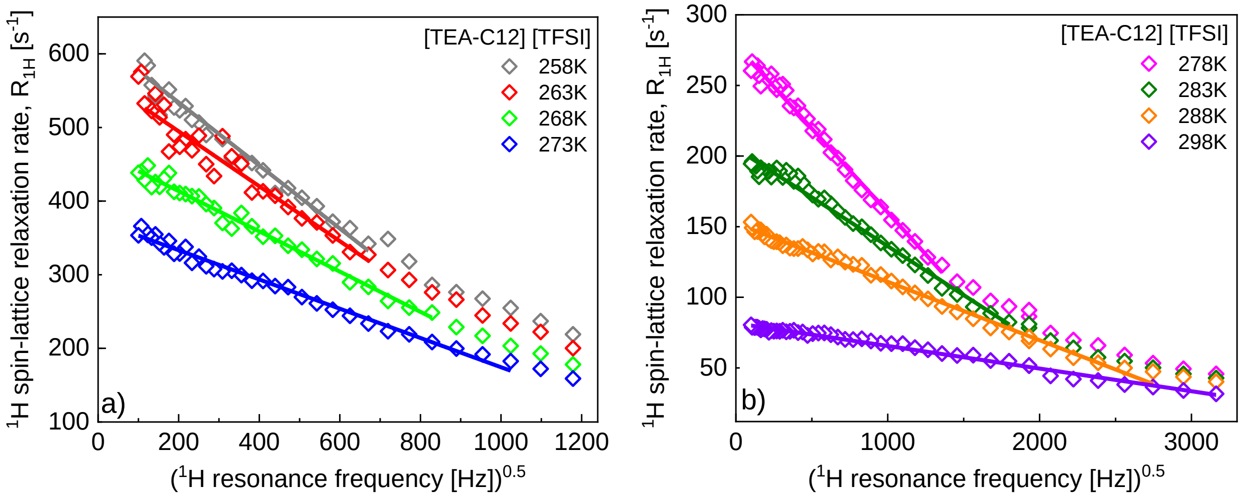

Figure S14a,b. [1]H spin-lattice relaxation data for [TEA-C12] [TFSI] versus squared root of resonance frequency; solid lines represent linear fits in the low frequency range.

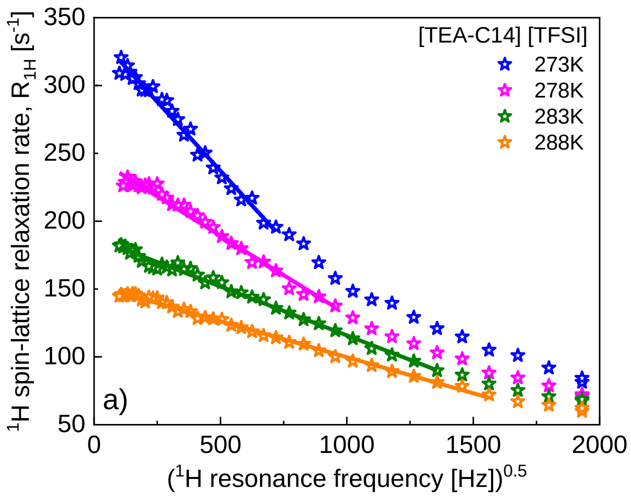

Figure S15a,b. [1]H spin-lattice relaxation data for [TEA-C14] [TFSI] versus squared root of resonance frequencies; solid lines represent linear fits in the low frequency range.

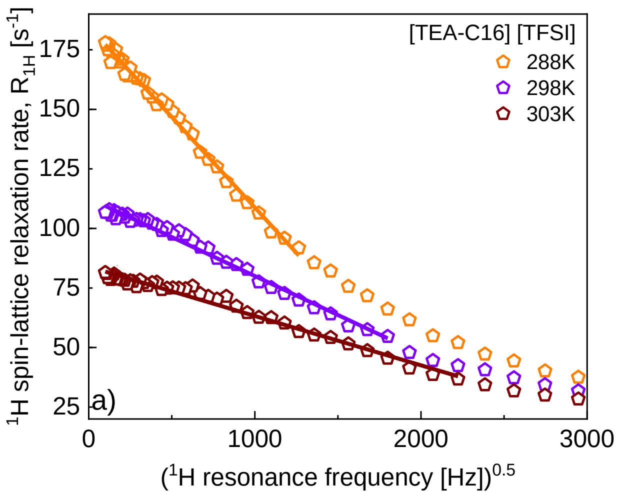

Figure S16a,b. [1]H spin-lattice relaxation data for [TEA-C16] [TFSI] versus squared root of resonance frequencies; solid lines represent linear fits in the low frequency range.

[1]H spin-lattice relaxation data decomposed into the inter-ionic, $R_{1,H}^{inter}(\omega)$, and intra-ionic, $R_{1,H}^{intra}(\omega)$, parts.

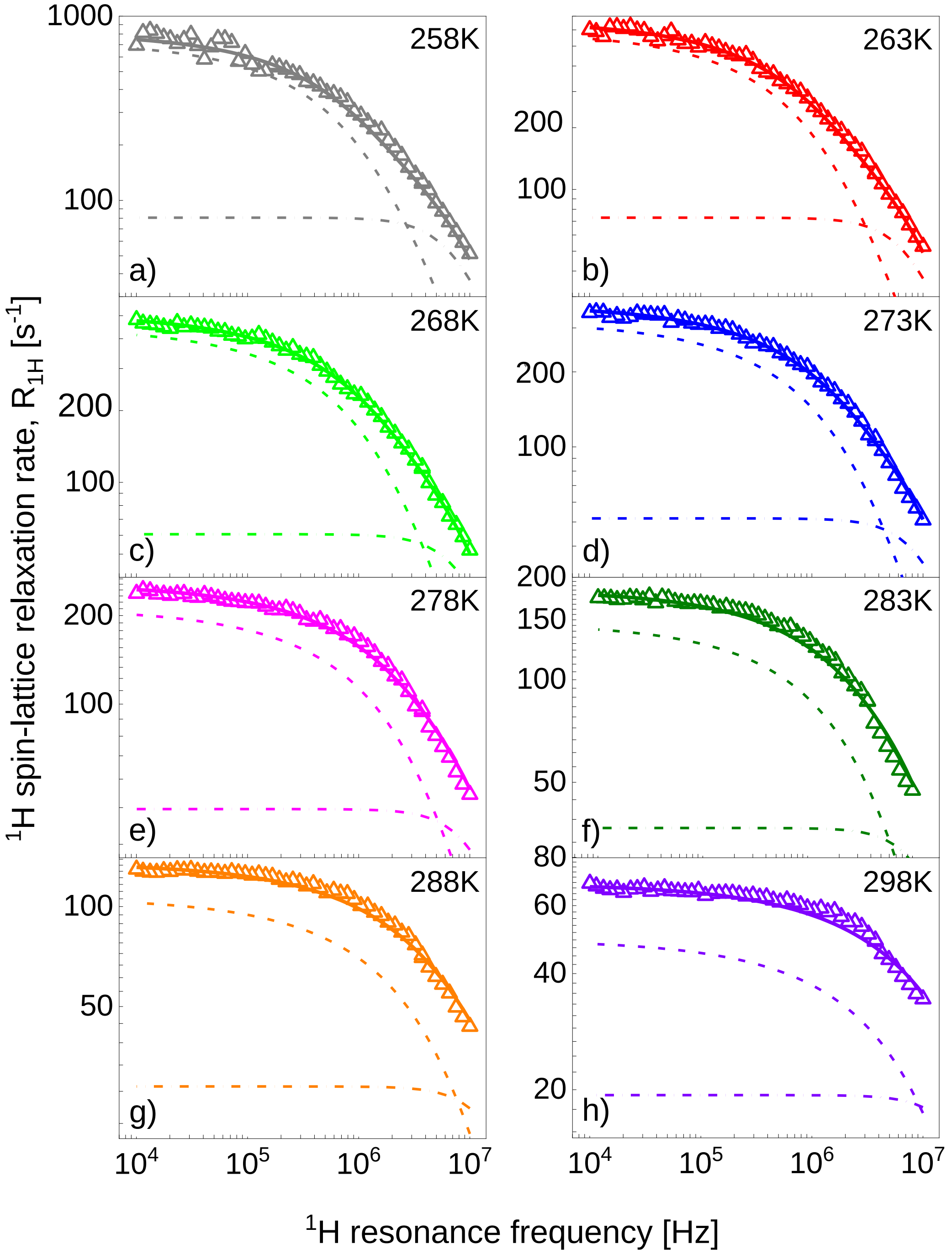

Figure S17. [1]H spin-lattice relaxation data for [TEA-C10] [TFSI]. Solid lines – fits decomposed into the inter-ionic, $R_{1,H}^{inter}(\omega)$, and intra-ionic, $R_{1,H}^{intra}(\omega)$, relaxation contributions represented as dashed and dashed-dotted lines, respectively.

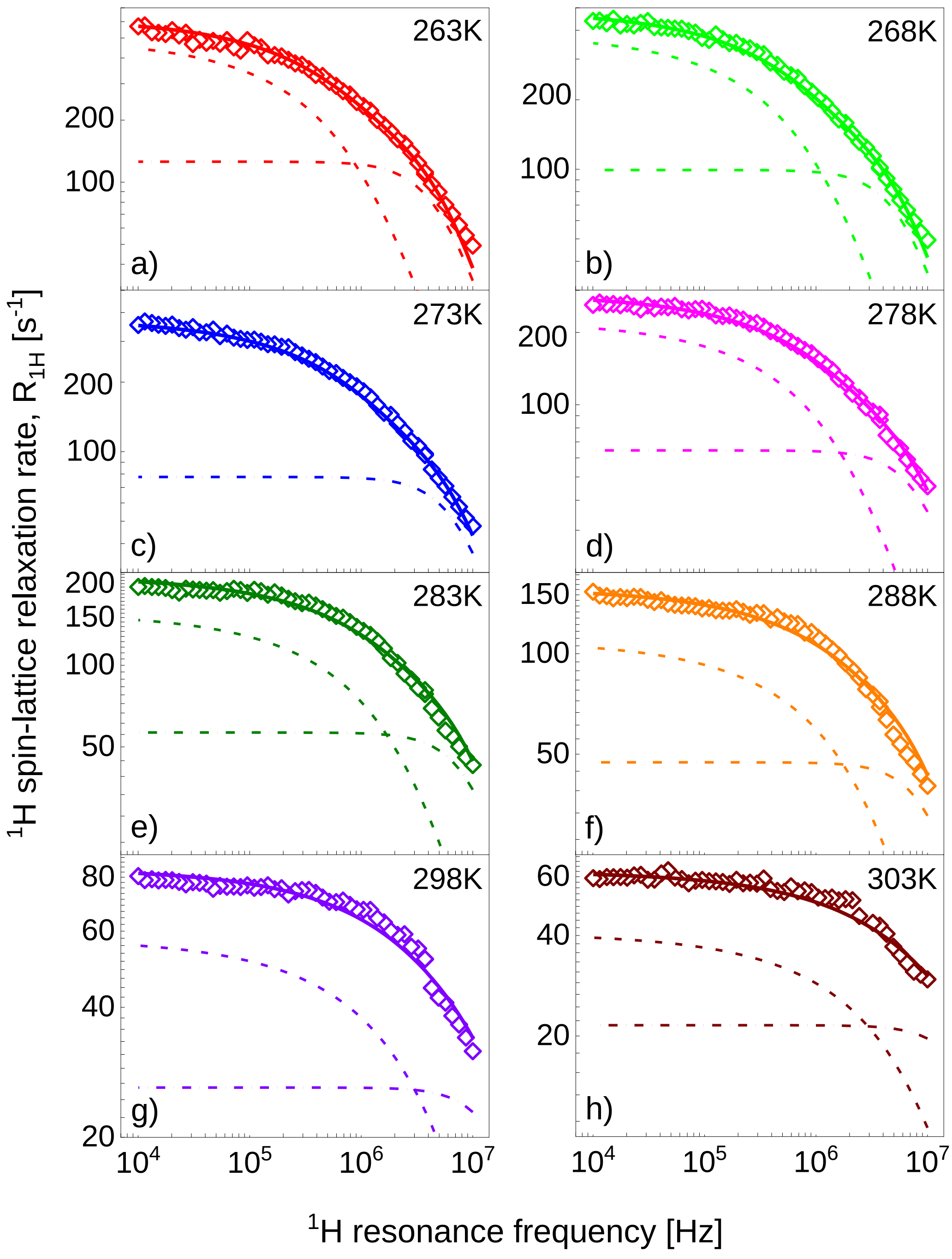

Figure S18. [1]H spin-lattice relaxation data for [TEA-C12] [TFSI]. Solid lines – fits decomposed into the inter-ionic, $R_{1,H}^{inter}(\omega)$, and intra-ionic, $R_{1,H}^{intra}(\omega)$, relaxation contributions represented as dashed and dashed-dotted lines, respectively.

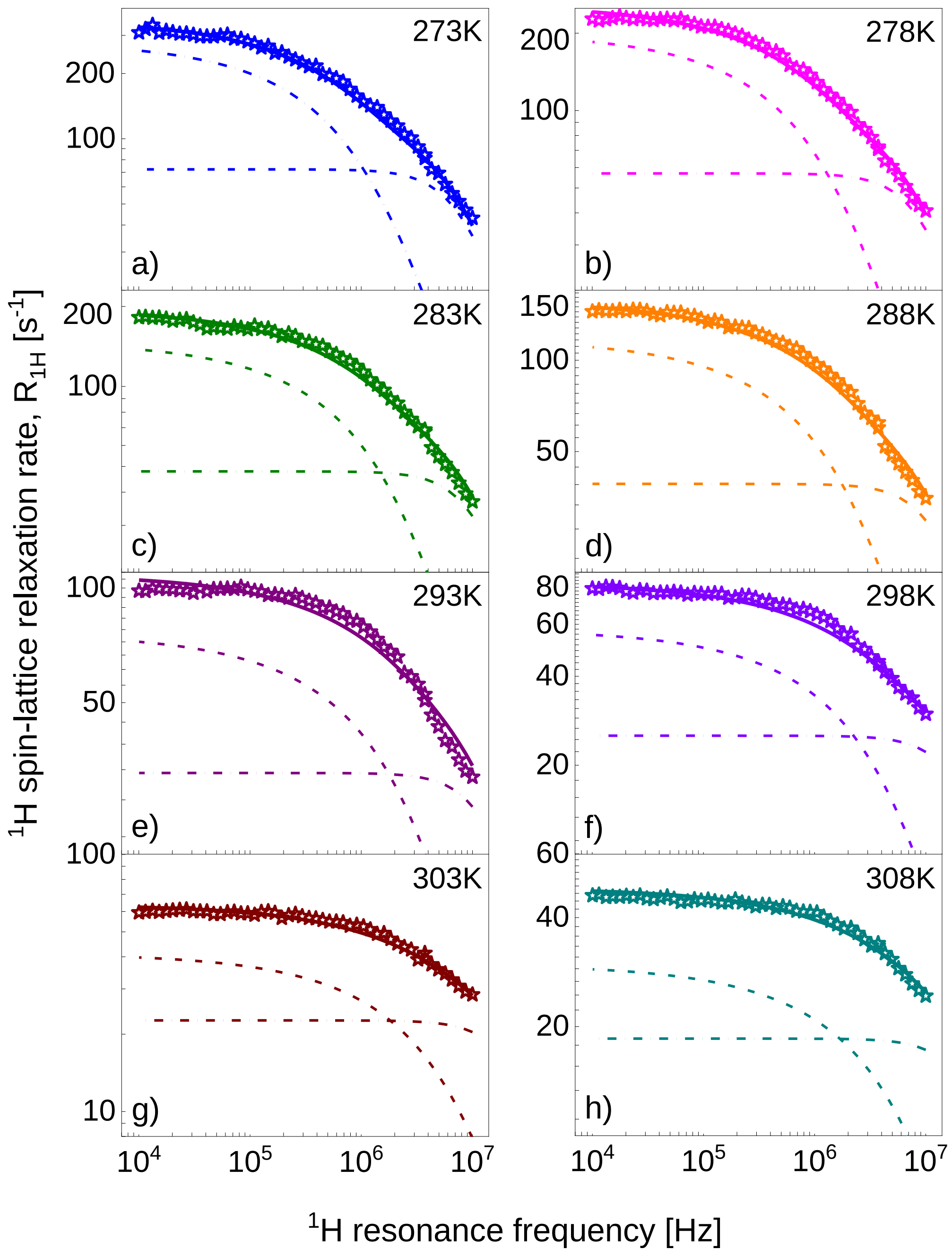

Figure S19. ^{1}H spin-lattice relaxation data for [TEA-C14] [TFSI]. Solid lines – fits decomposed into the inter-ionic, $R_{1,H}^{inter}(\omega)$, and intra-ionic, $R_{1,H}^{intra}(\omega)$, relaxation contributions represented as dashed and dashed-dotted lines, respectively.

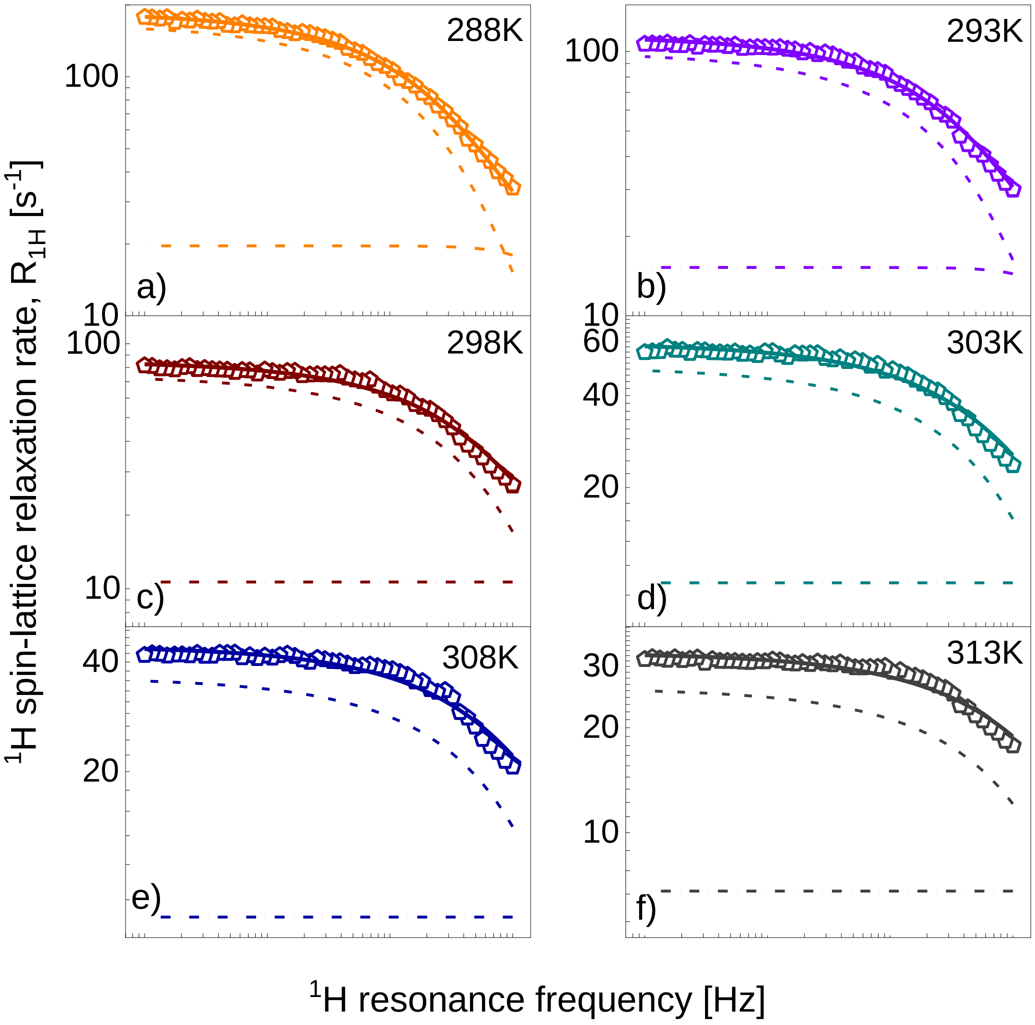

Figure S20. ^{1}H spin-lattice relaxation data for [TEA-C16] [TFSI]. Solid lines – fits decomposed into the inter-ionic, $R_{1,H}^{inter}(\omega)$, and intra-ionic, $R_{1,H}^{intra}(\omega)$, relaxation contributions represented as dashed and dashed-dotted lines, respectively.
